# A novel mitochondrial metabolism-related gene signature for predicting the prognosis of oesophageal squamous cell carcinoma

**DOI:** 10.18632/aging.205892

**Published:** 2024-06-05

**Authors:** Wenhao Lin, Changchun Ye, Liangzhang Sun, Zilu Chen, Chao Qu, Minxia Zhu, Jianzhong Li, Ranran Kong, Zhengshui Xu

**Affiliations:** 1 Department of Thoracic Surgery, The Second Affiliated Hospital of Xi’an Jiaotong University, Xi’an 710004, Shaanxi, China; 2 Department of General Surgery, The First Affiliated Hospital of Xi’an Jiaotong University, Xi’an 710061, Shaanxi, China; 3 Department of Thoracic Surgery, The First Affiliated Hospital of Wenzhou Medical University, Wenzhou 325000, Zhejiang, China; 4 Key Laboratory of Surgery Critical Care and Life Support (Xi’an Jiaotong University), Ministry of Education, Xi’an 710061, Shaanxi, China

**Keywords:** mitochondrial metabolism, oesophageal squamous cell carcinoma, prognosis, nomogram

## Abstract

Oesophageal squamous cell carcinoma (ESCC) is one of the most lethal cancers worldwide. Due to the important role of mitochondrial metabolism in cancer progression, a clinical prognostic model based on mitochondrial metabolism and clinical features was constructed in this study to predict the prognosis of ESCC. Firstly, the mitochondrial metabolism scores (MMs) were calculated based on 152 mitochondrial metabolism-related genes (MMRGs) by single sample gene set enrichment analysis (ssGSEA). Subsequently, univariate Cox regression and LASSO algorithm were used to identify prognosis-associated MMRG and risk-stratify patients. Functional enrichment, interaction network and immune-related analyses were performed to explore the features differences in patients at different risks. Finally, a prognostic nomogram incorporating clinical factors was constructed to assess the prognosis of ESCC. Our results found there were differences in clinical features between the MMs-high group and the MMs-low group in the TCGA-ESCC dataset (*P*<0.05). Afterwards, we identified 6 MMRGs (COX10, ACADVL, IDH3B, AKR1A1, LIAS, and NDUFB8) signature that could accurately distinguish high-risk and low-risk ESCC patients. A predictive nomogram that combined the 6 MMRGs with sex and N stage to predict the prognosis of ESCC was constructed, and the areas under the receiver operating characteristic (ROC) curve at 1, 2 and 3 years were 0.948, 0.927 and 0.848, respectively. Finally, we found that COX10, one of 6 MMRGs, could inhibit the malignant progression of ESCC *in vitro*. In summary, we constructed a clinical prognosis model based on 6 MMRGs and clinical features which can accurately predict the prognosis of ESCC patients.

## INTRODUCTION

Oesophageal squamous cell carcinoma (ESCC) is the predominant histological type of oesophageal cancer worldwide [1], accounting for approximately 85% of oesophageal cancer patients [2]. Although multimodality therapies, including surgery, chemotherapy, radiation therapy and molecular targeted therapy, are currently available for ESCC patients, the 5-year overall survival (OS) rate is still not satisfactory and remains in the range of 10%–30% in most countries [2–4]. Therefore, it is very important to identify a prognosis-related gene signature for ESCC and establish a prediction model for the individualized treatment of ESCC patients in the context of precision medicine.

Mitochondria are the center of oxidative phosphorylation and adenosine triphosphate (ATP) biosynthesis; ATP provides the majority of energy for mammalian cell biological processes [5]. Mitochondria also had a vital influence on the progression of malignant tumours due to their special position in energy metabolism [6]. Defective mitochondrial translation has been implicated in pathologies such as ageing, metabolic syndromes, and cancer [7]. Mitochondrial and metabolic pathway disorders caused by mitochondrial metabolism-related genes (MMRGs) promote tumour development, progression, and immune evasion [8, 9]. Previous research showed that MMRGs are strongly correlated with the malignancy of multiple tumours, such as pancreatic cancer, hepatocellular carcinoma, and acute myeloid leukaemia [10–14]. In ESCC, genes located in the mitochondrial inner membrane, such as the interferon-stimulating gene IFI6, are significantly overexpressed, which is related to the invasive phenotype and poor prognosis [9]. However, there is not enough data to identify the genetic characteristics related to MMRGs in ESCC and explore their impact on patient prognosis.

In the present study, we systematically analysed the expression levels, mutations and biological function of the 152 MMRGs in ESCC. In addition, we confirmed the key MMRGs in ESCC, and a prognostic risk score model based on the MMRGs signature and clinical features was constructed; the model successfully identified patients with higher prognostic risk.

## MATERIALS AND METHODS

### Data collection and processing

The count data, transcripts per million (TPM), and clinical data of the ESCC dataset were obtained from The Cancer Genome Atlas (TCGA) [15]. In total, 70 ESCC cancer samples with survival data and a final set of 18063 genes were included. The “Masked Somatic Mutation” data served as somatic mutation data [16], and the “Masked Copy Number Segment” data served as copy number variation (CNA) data; these data were visualized by R software. Tumour mutation burden (TMB) and microsatellite instability (MSI) were collected from the cBioPortal for Cancer Genomics (https://www.cbioportal.org/) [17]. GSE20347 (T=17, N=17, T means tumour and N means normal tissue) [18], GSE161533 (T=28, N=28), and GSE23400 (T=53, N=53) [19] were retrieved from the Gene Expression Omnibus (GEO) database [20] and merged to create a combined dataset for subsequent validation.

A total of 10 MMRGs were obtained from the GeneCards database [21] with relevance scores >2, and another 188 MMRGs were obtained from the KEGG PATHWAY database. Finally, 152 MMRGs were obtained by removing genes not found in the TCGA-ESCC dataset and the combined-dataset for subsequent analysis, as shown in Supplementary Table 1. All workflows are shown in Supplementary Figure 1.

### Calculation of the mitochondrial metabolism scores (MMs)

MMs in the merged dataset were calculated by the GSVA package [21] through the single sample gene set enrichment analysis (ssGSEA) algorithm. Then, the TCGA-ESCC dataset was grouped by the median of MMs, and the difference between the MMs-high and MMs-low groups was visualized by an accumulation map.

### Weighted gene co-expression network analysis (WGCNA)

The WGCNA package [22], with the settings of RsquaredCut to 0.85, the minimum number of module genes to 25, the module combined cutting height to 0, and the minimum distance to 0.2, was used to generate the co-expression module of TCGA-ESCC sample genes and MMs [11]. The R package clusterProfiler [23] was utilized to perform GO/KEGG [24, 25] analysis on the module genes with the largest positive and negative correlation, based on the standards of *P*.adjust<0.05 and FDR (*Q*.value) <0.20.

### Recognition of MMRG molecular subtypes

The patient samples from the TCGA-ESCC dataset were classified with the R package ConsonsusClusterPlus [26] according to the expression of MMRGs with unsupervised clustering. The number of clusters was set between 2 and 8, 1000 repeats were performed to extract 80% of the total samples, and clusterAlg= “pam” and distance= “euclidean” were run. Then, the infiltration of 28 tumour infiltration-associated immune cells was determined using the ssGSEA algorithm [27].

### Construction of the prognosis model based on MMRGs

Univariate Cox regression analysis was performed to identify the MMRGs related to OS with genes exhibiting *P*-values < 0.1. Subsequently, the significant variables were identified after eliminating multicollinearity through the LASSO algorithm. The risk score of each patient was calculated using the following formula.


$$\begin{array}{l} \text{riskScore}=\sum_{i}\;Coefficient\;(gene_{i})\\ \;\;\;\;\;\;\;\;\;\;\;\;\;\;\;\;\;\;\;\;*mRNA\;Expression\;(gene_{i}) \end{array}$$

Finally, the nomogram was constructed using R packet rms [28], and samples were divided into two groups based on the median risk score. To verify the stability and prediction ability of the model, decision curve analysis (DCA) [29], Kaplan-Meier (KM) curve and Receiver Operating Characteristic (ROC) curve were performed by R. The combined-dataset was used as a validation set for the same analysis as detailed above.

### Functional similarity analysis

The differentially expressed genes (DEGs) between the high- and low-risk groups were obtained using the DESeq2 package (*P*.adjust < 0.05 and |logFC| > 1). Then, GO/KEGG enrichment analysis for DEGs was performed. The GOSemSim package [30] was used to calculate the GO semantic similarity of genes, and the geometric mean of genes was calculated at the biological process (BP), cellular component (CC), and molecular function (MF) levels to obtain the final score. The ggplot package was used for visual analysis and visualization of the results.

### Identification and enrichment analysis of differentially expressed MMRGs

Gene Set Enrichment Analysis (GSEA) [31] is often used to evaluate the contribution of gene sets to functional phenotypes. The DEGs of TCGA-ESCC were sorted according to logFC and were enriched through the clusterProfiler package. Then, GSEA was conducted utilizing the following parameters: the seed number was 2020, which was calculated 1000 times, and each gene set contained at least 10 genes, with a maximum of 500 genes. We obtained the “c2.cp.all.v2022.1.Hs.symbols.gmt [All Canonical Pathways](3050)” gene set from the MSigDB database [32]. The significantly enriched screening criteria were *P*.adjust<0.05 and FDR value (*Q*.value) <0.20.

### Construction of the interaction network

The prediction of functionally similar genes among the selected key genes and the construction of an interaction network were carried out using the GeneMANIA website [33]. The prediction of miRNAs that interact with the key genes was carried out using the miRDB database [34], and an mRNA-miRNA interaction network was constructed for mRNAs with a target score > 80 using the miRDB database.

### Immune infiltration and variation analysis

We evaluated the immune cell infiltration status in the high- and low-risk groups using CIBERSORT (https://cibersort.stanford.edu/) [35] and calculated the relationships between various immune cells. The correlation between the key genes and immune infiltrating cells was determined, and a heatmap was generated for visualization using the R package “ggplot2”.

The somatic mutation data were pre-processed using VarScan software, and somatic mutations in the high-risk group were visualized using the maftools package [16]. The masked copy number segment data were downloaded using the R package TCGAbiolinks, and then GISTIC 2.0 analysis was conducted [36] through the Hiplot website (https://hiplot-academic.com/advance/gistic2).

### Construction of the clinical prognosis model

Based on TCGA-ESCC expression profile data, we used multivariate Cox regression, selected risk score combined with clinical features for Cox univariate analysis, and selected variable diseases with *P* < 0.1 to be included in the multivariate model. The predictive power of the model or a single variable was assessed by time-dependent ROC.

### Immunohistochemistry (IHC) and immunofluorescence

For IHC and immunofluorescence, the staining procedure was performed using the standard avidin–biotin complex method. Two pathologists evaluated all the specimens in a blinded manner. Five ESCC tissue samples and their paraneoplastic tissues were randomly selected from ESCC patients who had not received radiotherapy or chemotherapy before excision between May 2023 and August 2023. All patients underwent surgery at the Second Affiliated Hospital of Xi’an Jiaotong University. Informed consent was obtained for all patients. The details of the antibodies are presented in Supplementary Table 2.

### Cell culture and *in vitro* experiments

The KYSE140 (human oesophageal squamous cell carcinoma) cell line was maintained in RPMI-1640 medium supplemented with 10% FBS (Gibco BRL, Carlsbad, CA, USA) and 100 units/ml penicillin and streptomycin at 37° C in a humidified 5% CO_2_ atmosphere. Lentiviral infection was performed according to the manufacturer’s protocol as previously described [37]. The clone formation, migration and invasion assays were performed as previously described [38].

### Statistical analysis

All data processing and analysis were performed using R software (Version 4.1.2). For the comparison of two groups of continuous variables, the statistical significance of normally distributed variables was estimated through independent Student’s t test, and the difference between nonnormally distributed variables was analysed through the Wilcoxon rank-sum test. Comparisons with three or more groups were analysed using the Kruskal-Wallis test. The chi-square test or Fisher’s exact test was employed to compare and analyse the statistical significance of differences between two groups of categorical variables. The threshold for statistical significance was *P* < 0.05. In this study, ns stands for *P*≥0.05, * for *P* < 0.05, ** for *P* < 0.01, *** for *P* < 0.001, and **** for *P* < 0.0001.

## RESULTS

### Mitochondrial metabolism scores and SNP/CNV analysis of MMRGs

To understand the relationship between ESCC and mitochondrial metabolism, we calculated MMs for the TCGA-ESCC cohort based on 152 MMRGs. Afterwards, we demonstrated the differences in various clinical features (age, sex, T stage, N stage, M stage, and stage) between the high and low MMs groups divided by median MMs (Supplementary Figure 2A–2F). In the MMs-high group, the percentage of N2 and N3 was higher than those in the MMs-low group. Then we identified the mutations in 152 MMRGs (Supplementary Figure 2G) and the mutation waterfall plot of MMRGs was generated (Supplementary Figure 2H), with HTT having the highest mutation frequency. Finally, we analysed the copy number variation among the 152 MMRGs in ESCC (Supplementary Figure 2I). Results showed that EHHADH and NDUFB5 had the most copy number amplifications.

### Weighted co-expression network analysis

**Figure 1:**
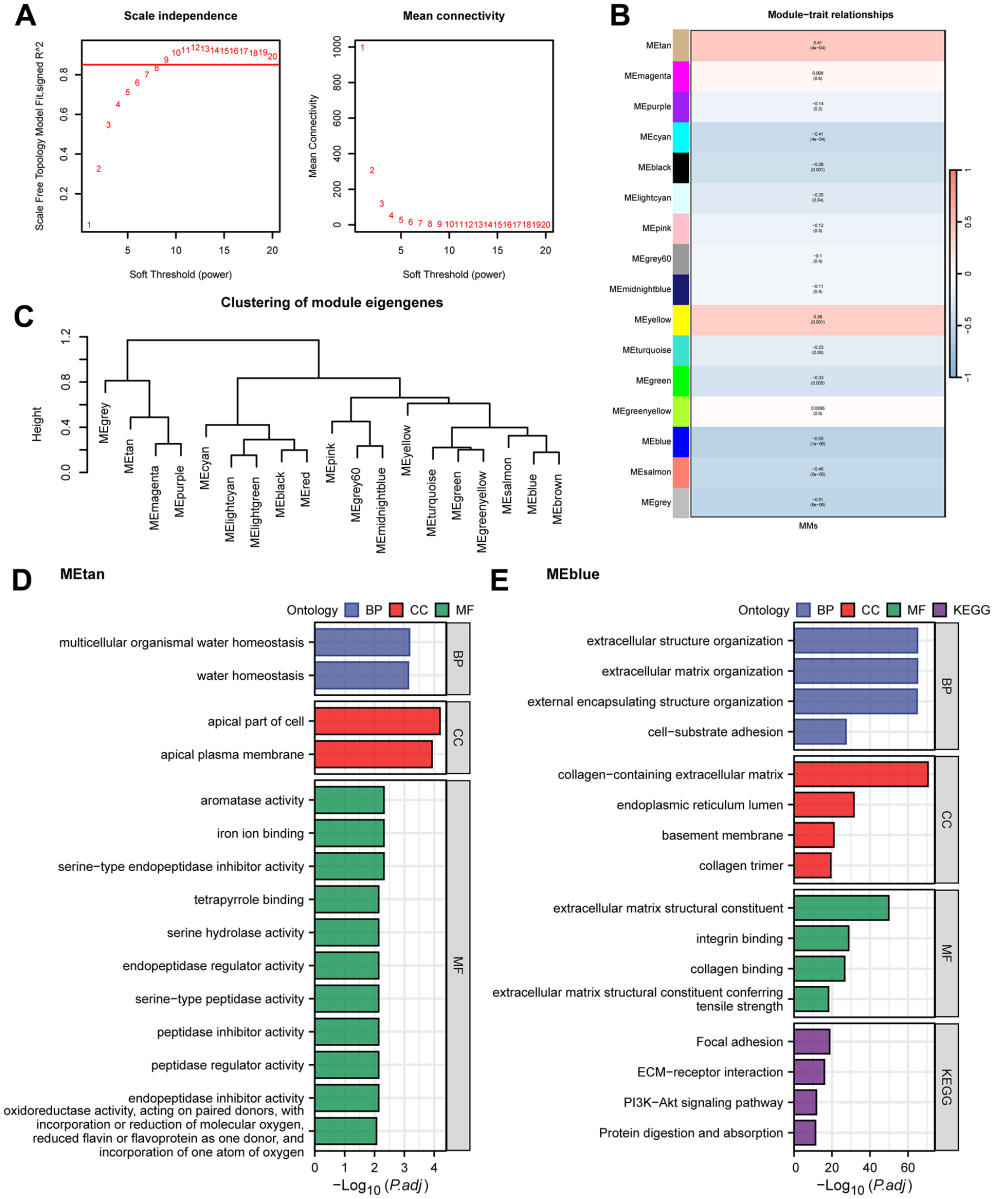
**WGCNA analysis and GO/KEGG analysis.** (**A**) WGCNA threshold screening graph. (**B**) The correlation heatmap between WGCNA module genes and MMs. (**C**) WGCNA module clustering tree. (**D**, **E**) GO/KEGG enrichment analysis of METan (**D**) module genes and MEblue (**E**) module genes.

To further identify the MMRGs closely related to ESCC, we conducted WGCNA on the TCGA-ESCC dataset to screen for co-expression modules. The analysis results showed that the optimal soft threshold was 9 (Figure 1A), and the genes in the TCGA-ESCC dataset were clustered into 16 modules (Figure 1B). Among them, METan had the highest positive correlation with MMs, with a correlation coefficient of R=0.41, while MEblue had the highest negative correlation with MMs (R=-0.55). The gene lists for METan and MEblue are shown in Supplementary Table 3, and the clustering of the module is shown in Figure 1C. Then, we conducted GO/KEGG analyses on the two modules with the highest correlation with MMs in the TCGA-ESCC dataset to explore their potential biological mechanisms. The 169 METan module genes were enriched in BPs, such as multiple organic water homeostasis; CCs, such as apical part of cell and apical plasma membrane; and MFs, such as oxidoreductase activity and incorporation of one atom of oxygen (Figure 1D and Supplementary Table 4). The 858 MEblue module genes were enriched in BPs, such as extracellular matrix organization and extracellular structure organization; CCs, such as collagen-containing extracellular matrix; and MFs, such as ECM-receiver interaction and the PI3K-Akt signalling pathway (Figure 1E and Supplementary Table 5).

### Unsupervised clustering of MMRGs

**Figure 2:**
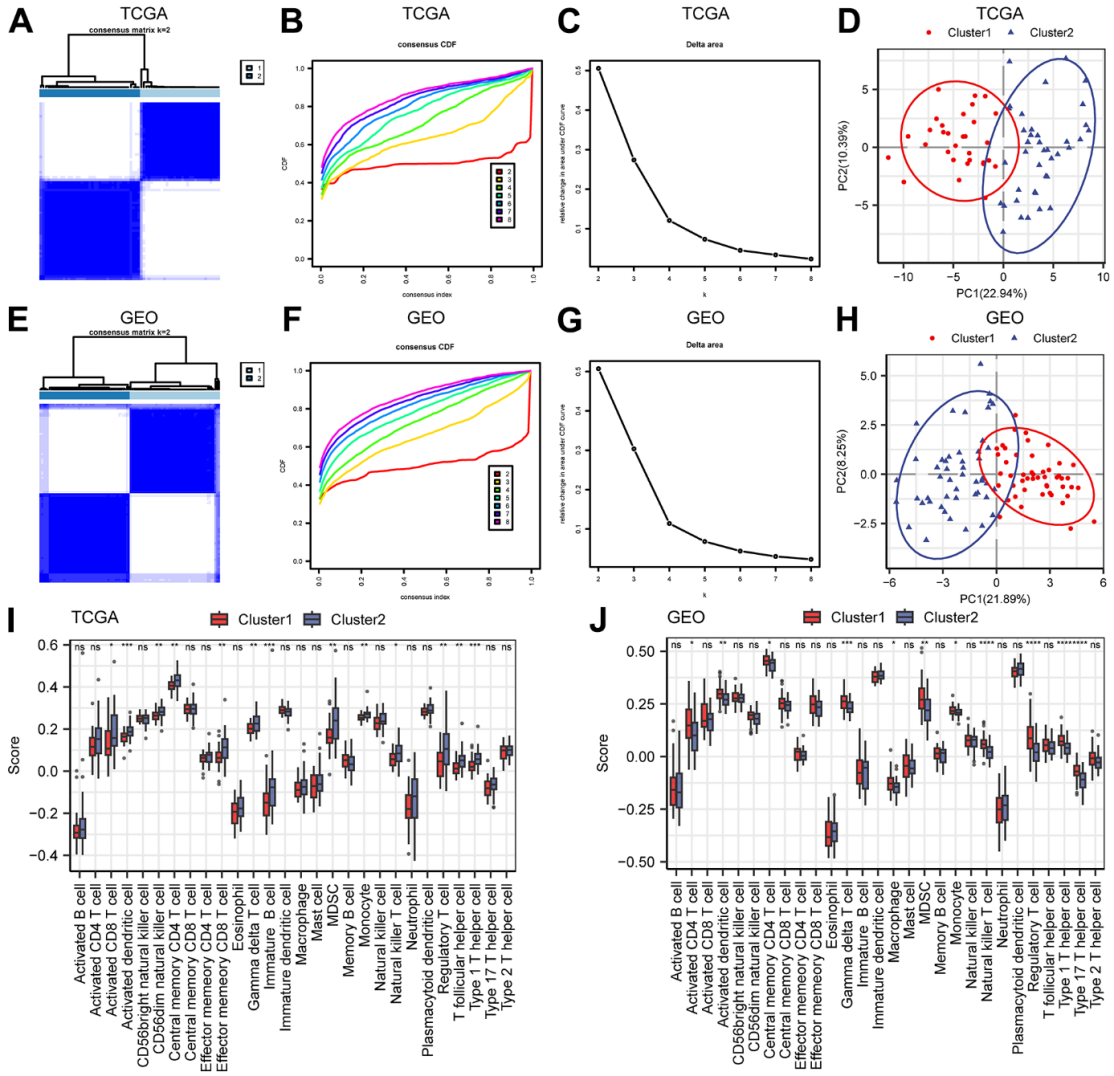
**Construction of molecular subtypes.** (**A**–**C**) The consistency clustering heatmap (**A**), consistency clustering cumulative distribution map (**B**), and consistency clustering Delta (**C**) of ESCC samples in the TCGA-ESCC dataset. (**D**) PCA diagram of TCGA-ESCC molecular subtypes. (**E**–**G**) Consistency clustering heatmap (**E**), consistency clustering cumulative distribution map (**F**), and consistency clustering Delta (**G**) of the merged GEO dataset. (**H**) PCA diagram of molecular subtypes in the merged GEO dataset. (**I**, **J**) Comparison of immune infiltration groups for different clusters in the TCGA-ESCC dataset (**I**) and the merged GEO dataset (**J**). Ns stands for P≥0.05, * for P<0.05, ** for P<0.01, *** for P<0.001, and **** for P<0.0001.

**Figure 3:**
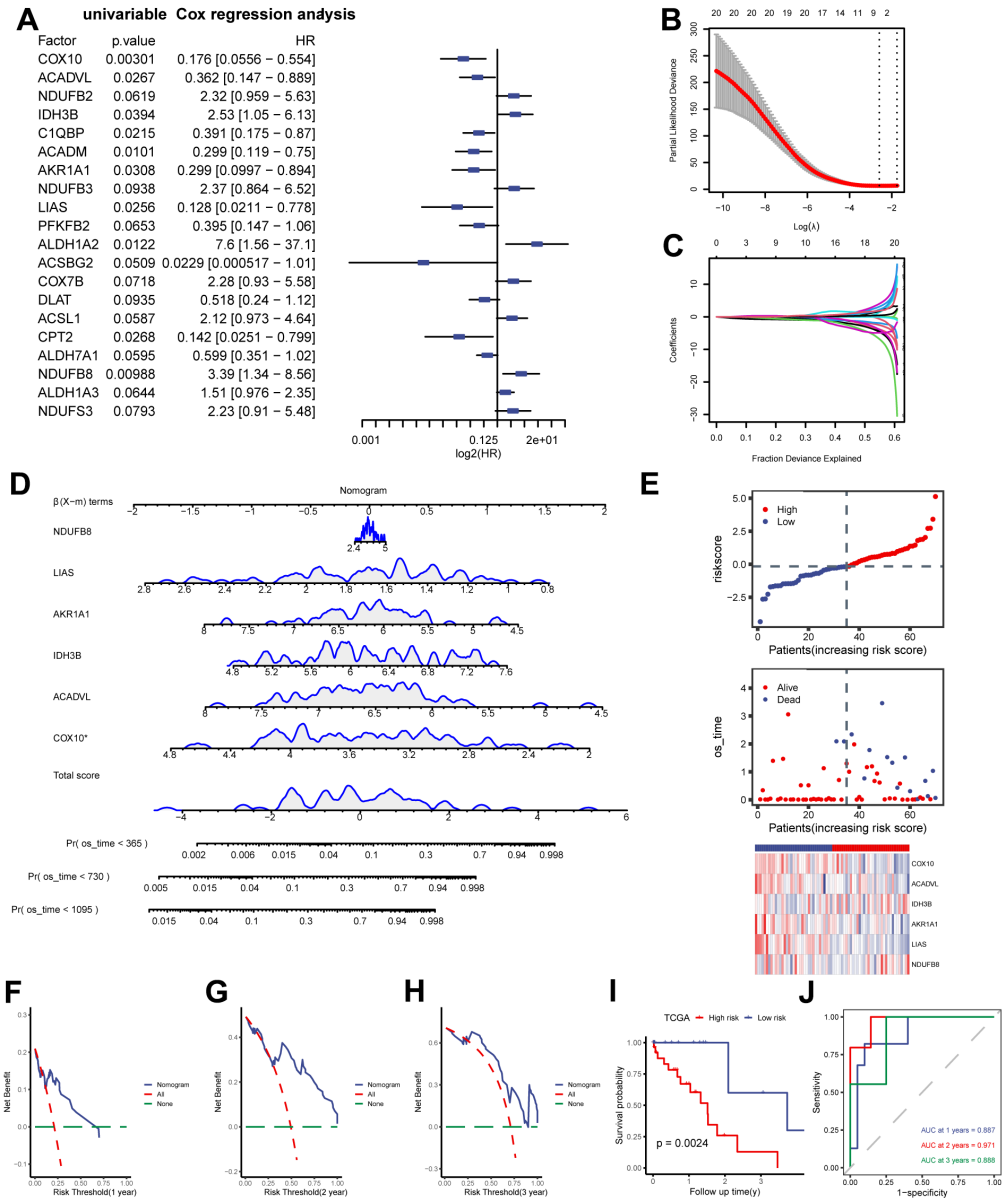
**Construction of MMRGs prognostic model.** (**A**) Cox unifactor analysis forest map of MMRGs. (**B**, **C**) LASSO regression analysis variable trajectory diagram (**B**), variable screening diagram (**C**). (**D**, **E**) MMRGs Cox Multifactor Analysis nomogram (**D**), Risk group map (**E**). (**F**–**H**) Prognosis DCA map of Cox multivariate model at 1, 2, 3 years. (**I**, **J**) The KM curve (**I**) and ROC (**J**). MMRGs: Mitochondrial metabolism-related genes. LASSO: Least absolute shrinkage and selection operator. DCA: Decision curve analysis.

To better characterize the heterogeneity of mitochondrial metabolism in ESCC patients, we used the expression data of 152 MMRGs to perform unsupervised clustering of all ESCC patient samples based on a consensus clustering algorithm, which was used to identify the corresponding molecular subtypes. The results suggested that when the optimal number of clusters was 2, the cluster effect was the best (Figure 2A–2C). Therefore, we clustered all samples into two clusters (Cluster1=31 and Cluster2=39). Principal component analysis (PCA) showed that all patients were roughly divided into two groups, confirming the stability of this clustering (Figure 2D). To further validate the reliability of this clustering approach, we also included a merged dataset of the 3 GEO datasets as an external validation. The batch effects of the 3 datasets were removed in the merger (Supplementary Figure 3A–3D). We conducted the same analysis on the combined-dataset, and this dataset was also divided into two categories (Cluster1=49 and Cluster2=49, Figure 2E–2H). Subsequently, we performed immune infiltration analysis using two types of samples from the TCGA-ESCC and combined datasets (Figure 3I, 3J). In the two datasets, immune cells, such as central memory CD4 T cells, gamma delta T cells, MDSCs, and monocytes, had significant infiltration differences in different subtypes. This result indicated that MMRGs can characterize ESCC samples into two different subtypes based on mitochondrial metabolism.

### Construction and prognostic analysis of MMRGs models

Based on the previous analysis, we quantified the impact of MMRGs on the prognosis of each ESCC patient and constructed a risk model by integrating the expression of 152 MMRGs. Initially, 20 prognosis-related MMRGs were identified using univariate Cox regression with TCGA-ESCC (Figure 3A). Subsequently, LASSO regression was used to eliminate the collinearity of these 20 genes, determine the best lambda value and construct cross validation (Figure 3B, 3C). Finally, we identified 6 prognostically relevant key genes (COX10, ACADVL, IDH3B, AKR1A1, LIAS, and NDUFB8), and based on these 6 genes we mapped the nomogram of prognostic risk (Figure 3D). According to the median value of the risk score, ESCC patients were divided into high- and low-risk groups (Figure 3E). The DCA curves demonstrated the good predictive ability of the Cox model for 1-, 2-, and 3-year survival risk in ESCC patients (Figure 3F–3H). As expected, the K-M curve showed that patients in the high-risk score group had a worse prognosis than those in the low-risk score group (Figure 3I), and ROC also indicated a good predictive ability, with the highest predictive performance at 2 years (AUC=0.971, Figure 3J).

### Comparative analysis between high and low risk groups

**Figure 4:**
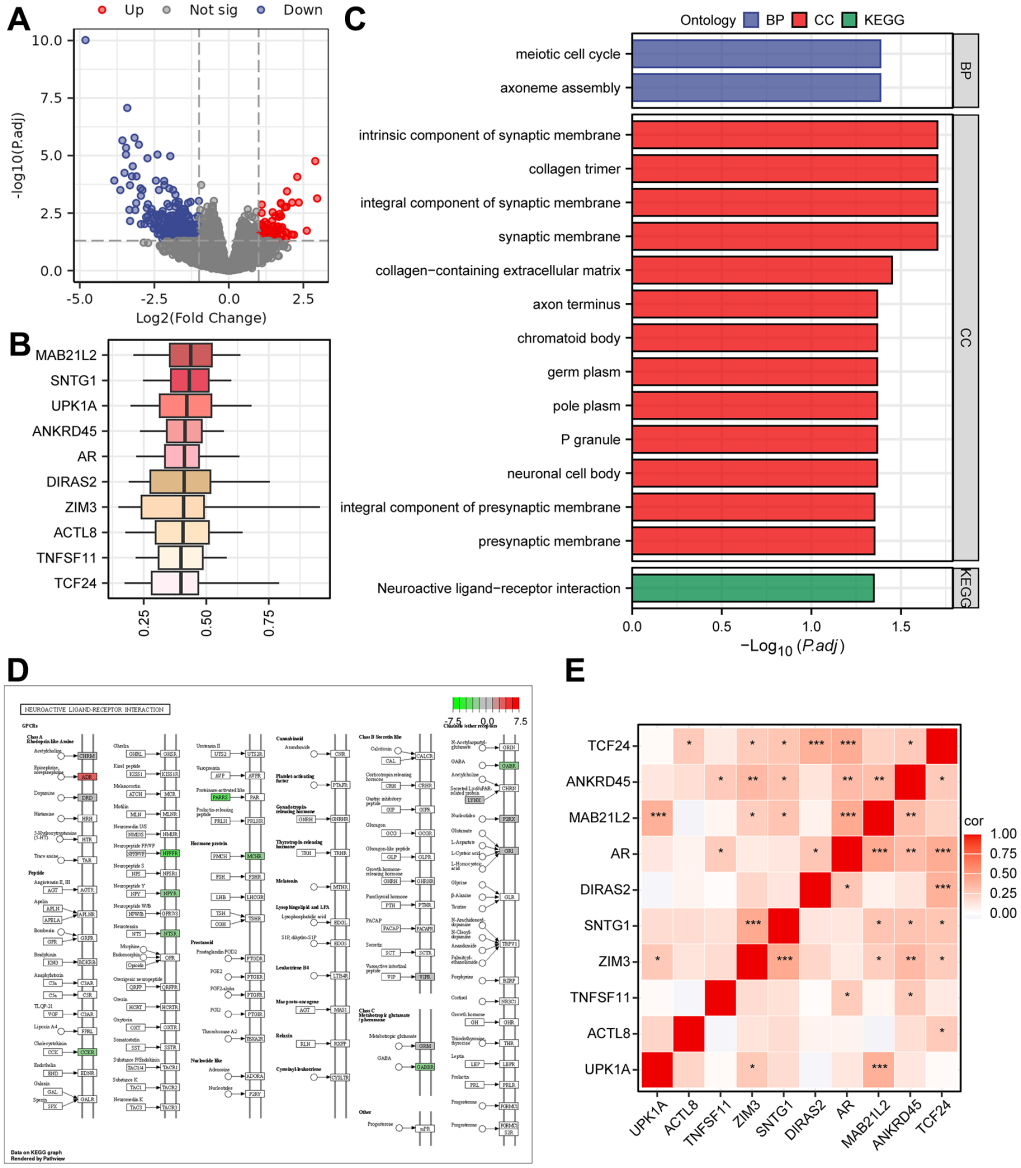
**The results of Gene Ontology (GO) and KEGG pathway analyses.** (**A**) Volcano plot of DEGs grouped as high- and low-risk using the Cox regression model. (**B**) Box plots showing the functional similarity analysis of the top 10 genes. (**C**) Bar plots displaying the results of GO and KEGG pathway enrichment analyses of the DEGs. (**D**) Pathway map for the Neuroactive ligand-receptor interaction pathway, with color mapping from green to red indicating increasing logFC values. (**E**) Correlation heatmap for the top 10 functionally similar genes.

To identify DEGs between the two groups of patients, we conducted differential analysis, and a total of 399 genes (*P*.adjust < 0.05 and |logFC| > 1) were identified (Supplementary Table 6 and Figure 4A). Furthermore, functional similarity analysis was performed, and the top 10 DEGs were MAB21L2, SNTG1, UPK1A, ANKRD45, AR, DIRAS2, ZIM3, ACTL8, TNFSF11, and TCF24 (Figure 4B). The results of the GO enrichment analysis demonstrated that BPs, such as axoneme assembly and meiotic cell cycle, and CCs, such as integral component of synaptic membrane and collagen trimer, were significantly enriched (Figure 4C). Additionally, the neuroactive ligand-receptor interaction pathway in KEGG was enriched (Supplementary Table 7 and Figure 4C, 4D). We also generated a correlation heatmap of the top 10 functionally similar genes, which showed that most of these genes exhibited significant positive correlations (Figure 4E). To further determine the influence of DEG expression between the two risk groups, we analysed the correlation between DEG and enriched pathways through GSEA (Supplementary Figure 4A and Supplementary Table 8). The results revealed that DEGs were significantly enriched in the IL 18 signalling pathway (Supplementary Figure 4B), oxidative phosphorylation (Supplementary Figure 4C), metabolism of polyamines (Supplementary Figure 4D), electron transport chain OXPHOS system in mitochondria (Supplementary Figure 4E), and electron transport (Supplementary Figure 4F and Supplementary Table 8).

### Analysis of the interactions among key genes

**Figure 5:**
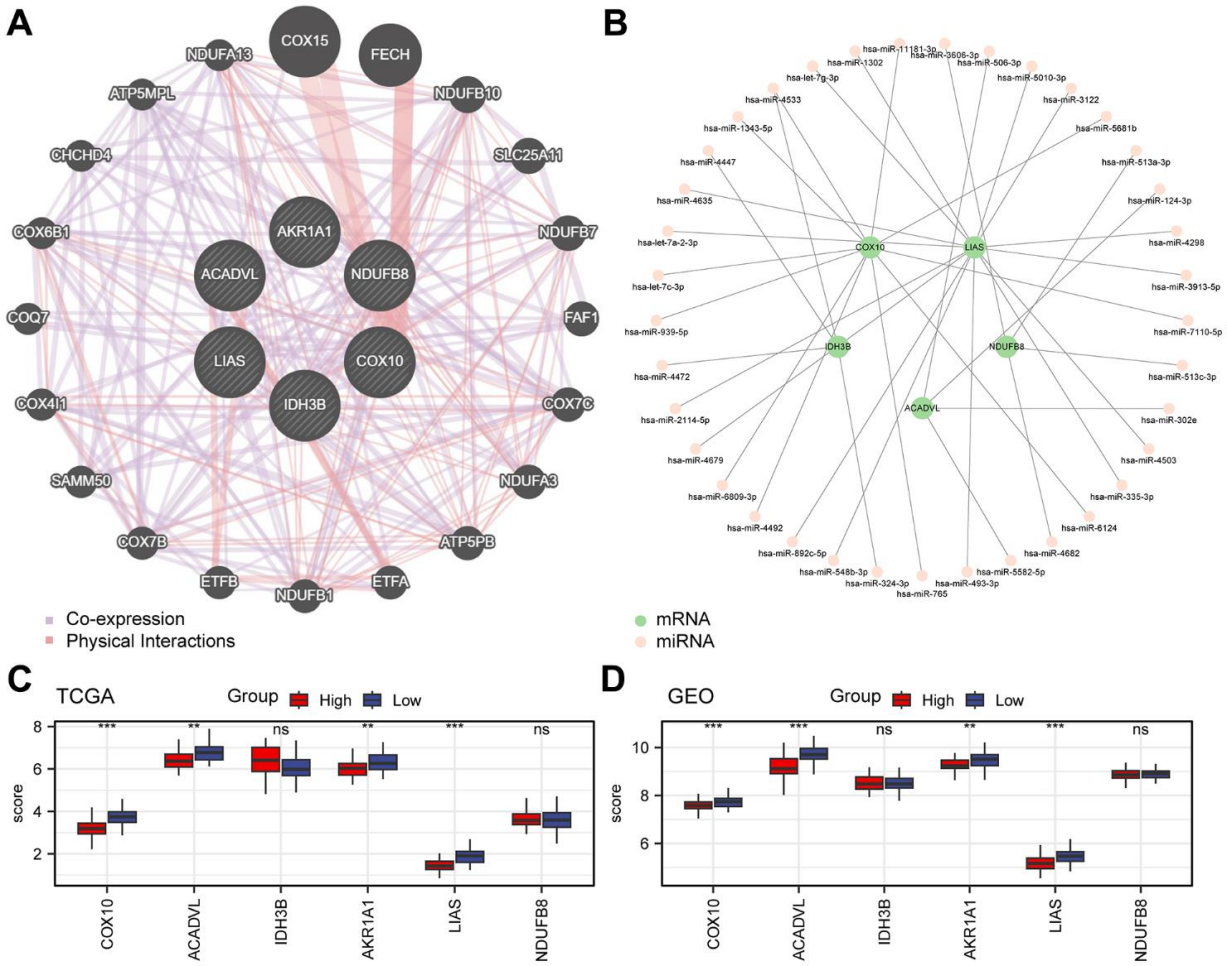
**Analysis of key genes.** (**A**) Interaction network of functionally related genes of key genes in the GeneMANIA website. (**B**) Network diagram of mRNA-miRNA. The green nodes represent the key genes (mRNA), and the pink nodes represent miRNA. (**C**, **D**) Grouped comparative graphs of high- and low-risk groups of key genes in the TCGA-ESCC dataset (**C**) and the combined GEO dataset (**D**).

To understand the wide range of associations among biomolecules, we used the GeneMANIA website to further analyse genes functionally associated with the 6 prognostically relevant key genes and constructed the interaction networks among them (Figure 5A). In addition, there is also a complex regulatory relationship between miRNAs and gene expression, so we predicted the mRNA-miRNA interaction network of these 6 genes with the help of miRDB database (Figure 5B). The network consisted of 5 key genes (COX10, ACADVL, IDH3B, LIAS, and NDUFB8), 37 miRNA molecules, and 38 pairs of mRNA-miRNA interactions in total (Supplementary Table 9). Further, we analyzed the expression of these six genes in two risk subgroups (Figure 5C, 5D). Results revealed that COX10, ACADVL, AKR1A1, and LIAS exhibited significant expression differences and consistent expression trends in both TCGA-ESCC and combined-GEO datasets (*P* < 0.05).

### Immune infiltration analysis

To understand the differences in immune cell infiltration in the tumour microenvironment between the two groups in the Cox model, infiltration was calculated using CIBERSORT with the TCGA-ESCC dataset (Supplementary Figure 5A, 5D). The correlation heatmap revealed that most immune cells were significantly correlated with activated mast cells and M0 macrophages (Supplementary Figure 5B). Additionally, correlation analysis of key genes also demonstrated that AKR1A1 is associated with the largest number of immune cells out of all the key genes (Supplementary Figure 5C).

### SNP and CNV analysis

To compare mutations in the high- and low-risk groups in the TCGA-ESCC dataset, we generated a mutation waterfall plot, which demonstrated that TP53 exhibited the highest mutation frequency (Supplementary Figure 6A). Additionally, we conducted GISTIC 2.0 analysis on the CNV segments of the two groups (Supplementary Figure 6B–6E). The CNV analysis results revealed that the largest increase in the number of mutated CNVs was observed at 11q13.3, while the largest decrease in the number of CNVs occurred at 9q21.3 in both groups.

### Construction of the clinical prognosis model based on the risk score

**Figure 6:**
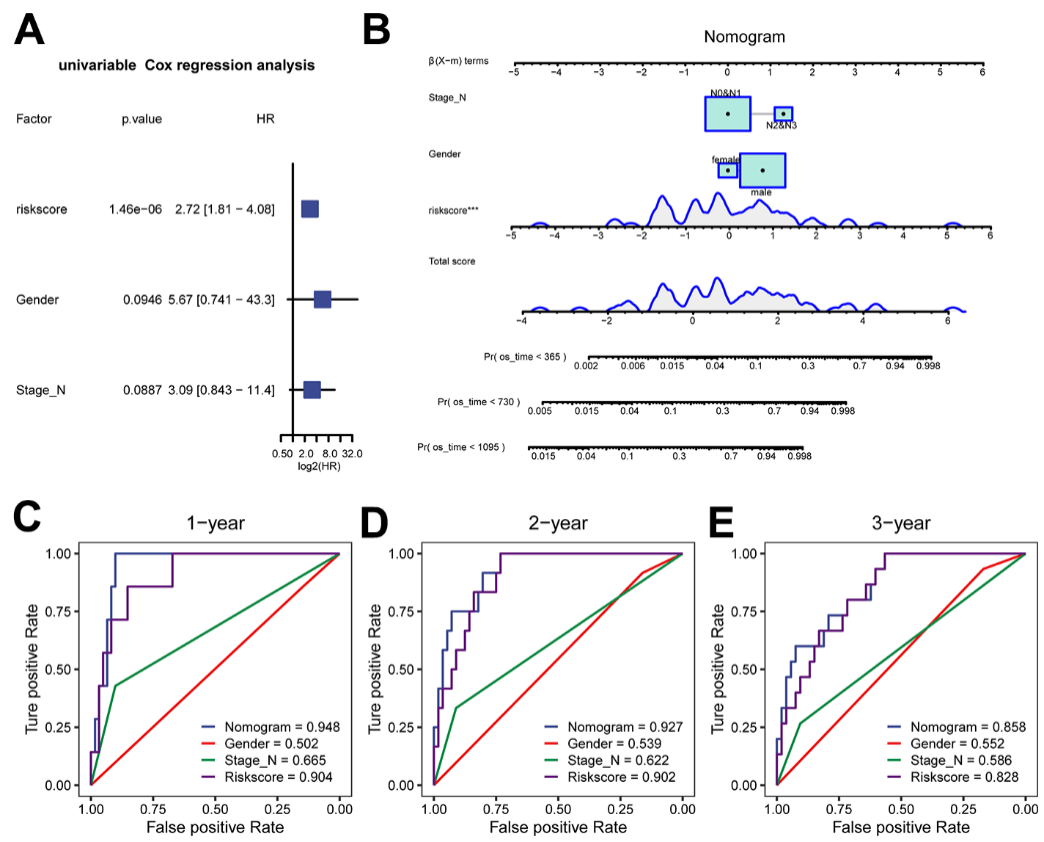
**Clinical prediction model based on risk score in the TCGA-ESCC dataset.** (**A**) Forest plot of univariate analysis for the clinical prediction model. (**B**) Nomogram of the clinical prediction model for multivariate analysis. (**C**–**E**) ROC curves of the clinical prediction model for 1 year (**C**), 2 years (**D**), and 3 years (**E**) compared to single factors.

To improve the clinical predictability of the model, we constructed a predictive nomogram by combining the Cox risk score model with clinical characteristics. Firstly, we compared the differences in various clinical factors between the two groups of the Cox model (Supplementary Figure 7A–7F). Then univariate Cox analysis indicated that the risk score, sex, and N stage had *P*-values less than 0.1 and were included in the final model (Figure 6A). Finally, a nomogram was constructed, which could be utilized to determine the probability of survival for less than 1, 2 or 3 years (Figure 6B). To further evaluate the performance of the nomogram in comparison to that of other single factors, we plotted ROC curves within 3 years (Figure 6C–6E). The results suggested that the AUC values exceeded those of the other single factors and indicated that the nomogram had better discriminative ability compared to single factors.

### The expression of 6 MMRGs in tissue samples and the role of the COX10 protein *in vitro*

**Figure 7:**
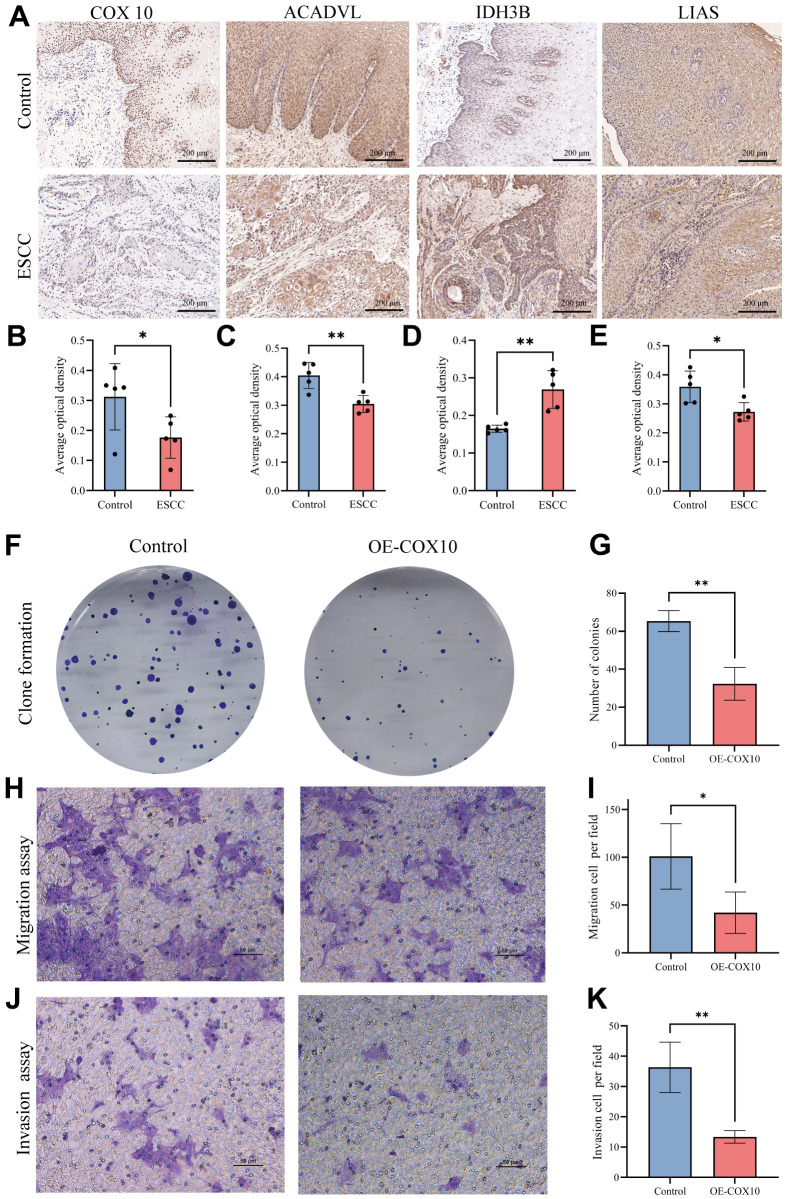
**The expression of MMRGs and the role of COX10 protein in ESCC.** (**A**) Representative image of the IHC staining of COX10, ACADVL, IDH3B and LIAS. (**B**–**E**) Quantitative statistics of the expression of COX10 (**B**), ACADVL (**C**), IDH3B (**D**) and LIAS (**E**). (**F**) Colony formation of ESCC cells transfected with COX10 or vector. (**G**) Quantitative statistics of the Colonies. (**H**) The migration ability of ESCC cells transfected with COX10 or vector. (**I**) Quantitative statistics of the migration cells per field. (**J**) The invasion ability in ESCC cells transfected with COX10 or vector. (**K**) Quantitative statistics of the invasion cells per field. Each experiment was repeated three times independently, and * stands for *P*<0.05, ** stands for *P*<0.01.

To further understand the role of the 6 prognostically critical genes in ESCC in our model. We first analysed their expression in ESCC tissues and paracancerous tissues with the help of IHC. The results showed that COX10, ACADVL, IDH3B, and LIAS were significantly differentially expressed in ESCC tissues (Figure 7A–7E). Considering that COX10 had the smallest *P*-value in univariate and multivariate Cox regression analyses (Figure 3A and Supplementary Figure 8), we paid particular attention to this gene. Immunofluorescence results confirmed COX10 expression in mitochondria (Supplementary Figure 9). After overexpression of COX10 using the lentiviral, the clone formation ability of KYSE140 cells was significantly inhibited (Figure 7F, 7G). In addition, transwell experiments showed that the migration and invasion abilities of KYSE140 cells were also significantly inhibited upon overexpression of COX10 (Figure 7H–7K). These results suggest that COX10 indeed plays an important role in inhibiting the malignant progression of ESCC.

## DISCUSSION

ESCC is among the deadliest malignant tumours with poor prognosis; however, there is still no ideal method to predict the prognosis of ESCC to date [2–4]. Although the TNM staging system is updated periodically, it does not predict the prognosis of ESCC patients accurately. In recent years, an increasing number of studies have begun to highlight the enormous potential of mRNA as a molecular marker for predicting ESCC patient prognosis [39]. Considering that the development of ESCC is a complex clinical pathological process with genetic heterogeneity, a predictive model that integrates multiple factors may better assess the outcome of patients than a single biomarker.

Mitochondria are central to cell energy metabolism [5], and dysregulation of cellular energy metabolism is a significant characteristic of tumours [40]. Mitochondrial metabolism is involved in the malignant transformation of cells, tumour progression, therapeutic response and immune monitoring [41]. In ESCC, recent research has shown that circPUM1 localized in mitochondria and regulated the oxidative phosphorylation in cancer cell mitochondria to enhance the tumorigenicity of ESCC cells *in vivo* and *in vitro* [42]. Similarly, researchers have found that STAT3β can disrupt the electron transport chain of mitochondria and enhance the chemosensitivity of ESCC cells [43]. Therefore, in this study, we demonstrated the heterogeneity of mitochondrial metabolism in ESCC patients and identified a gene signature of MMRGs related to ESCC. Finally, we constructed a more reliable clinical prediction model by combining the clinical characteristics of patients and the 6-gene prognostic signature.

In our study, we identified co-expression modules of ESCC genes through WGCNA and found a significant correlation between ESCC modules and MMs. Among them, the genes in the modules with the strongest positive and negative correlation (r=0.41 and r=-0.55) were significantly enriched in mitochondrial metabolism, and specifically oxidoreductase activity acting on paired donors with incorporation or reduction of molecular oxygen. Furthermore, based on MMRGs, all patients can be divided into two subtypes through unsupervised clustering, which further confirms the important role of mitochondrial metabolism in ESCC, as was reported in previous studies. Notably, the pattern of immune infiltration varied between the two subtypes of patients; this variation is consistent with the results showing that mitochondrial metabolism is involved in cancer cell immune monitoring. For example, the infiltration of MSDC in immunosuppressive cells showed significant differences in the two subtypes (p<0.001 in TCGA and p<0.05 in GEO), and mitochondrial oxidative phosphorylation could promote the differentiation of MSDC and drive its immunosuppressive function [41, 44]. The infiltration scores of some immune cells in ESCC differed somewhat between the TCGA-ESCC and combined-GEO dataset due to the technical platform differences and biological variability between the sample sources, but this did not affect our conclusion that the immune microenvironment of ESCC with different mitochondrial metabolic states differed. More specific studies are still needed in the future to explore the complex crosstalk between mitochondrial metabolism and tumour immunity. In summary, MMRGs have remarkable potential in the prediction of ESCC patient prognosis.

The use of gene signatures to construct predictive models is a novel research method for identifying tumour prognostic biomarkers. Song et al. developed an immune signature to evaluate the outcomes of lung cancer patients [45], and Tong et al. similarly developed mitochondrial metabolism-related gene signatures for acute myeloid leukaemia [46]. For ESCC, a 5-gene prognostic signature based on m6A RNA methylation and a 10-genes related to ferroptosis was constructed, and the prediction performance was good, with the best accuracy of approximately 75% [47, 48]. However, the prediction of patient prognosis based on these gene signatures often lacks clinical correlation. In the present study, we found differences in multiple clinical features of ESCC patients in the high and low MMs groups, such as age, sex and N stage. After constructing a model based on our MMs signature, we conducted univariate Cox analysis to identify the clinical characteristics related to prognosis risk (sex and N stage) and included them in our model. Finally, a clinical prediction model was constructed. Remarkably, the MMRG signature combined with clinical features could predict the outcomes of ESCC patients in 3 years with AUCs of 0.948, 0.927, and 0.858, which are generally higher than those of previous models (0.600 in the m6A RNA signature [47] and 0.751 in the ferroptosis-related signature [48]). Altogether, we built a novel clinical prediction model for ESCC based on the MMRG signature, and the predictive value was generally higher than that of previous predictive models.

To identify the MMRGs gene signature for prognostic models, we systematically investigated the 152 MMRGs in patients with ESCC. Finally, 6 core genes (COX10, ACADVL, IDH3B, AKR1A1, LIAS, and NDUFB8) were identified through the LASSO regression algorithm and included in our MMRGs signature for multivariable Cox regression. Notably, most of these key genes are reportedly involved in various cancers. ACADVL variants result in long-chain acyl-CoA dehydrogenase deficiency in mitochondria [49], while recent research indicates that tumour-specific T cells can be metabolically reprogrammed via the forced expression of ACADVL, which promoted the survival of tumour-specific T cells in a pancreatic cancer mouse model and improve their immunotherapeutic effects [50]. ACADVL overexpression is important to leukaemia mitochondrial metabolism because the loss of ACADVL activity results in the repression of cell proliferation, clonogenic potential, and engraftment in leukaemia cells [51]. However, in our study, higher expression of ACADVL was associated with better outcomes in ESCC. The opposite effects of ACADVL might be driven by the different tumour microenvironments in solid tumours and haematologic tumours. IDH3β is considered a novel APC/C-CDH1 substrate and an important regulator of the cell cycle that can promote cell proliferation in ESCC, and the overexpression of IDH3β is often correlated with poor prognosis in ESCC [52]; this correlation is consistent with the high HR of IDH3β in our study (HR=2.29). AKR1A1 is a member of the human aldo-keto reductase (AKR) family, which is widely distributed in most cancer cells with relatively stable abundances [53]. AKR1A1 expression increases following radiation of laryngeal cancer, thereby inhibiting the activation of p53; thus, AKR1A1 plays a role in acquired radiation resistance in laryngeal cancer cells [54]. Variant LIAS could result in defective mitochondrial metabolism [55]. High LIAS expression has been correlated with a better prognosis in multiple cancer patients, such as kidney carcinoma, rectum adenocarcinoma and breast cancer [56], and this correlation is consistent with our findings. NDUFB8 is a subunit of mitochondrial complex I, and the inhibition of NDUFB8 can mediate excessive ROS production and ATP depletion, which may induce apoptosis in gastric adenocarcinoma cells [57]. Notably, both our univariate and multivariate regression analyses indicated that COX10 is a key gene in mitochondrial metabolism in ESCC.COX10 promotes the assembly of mitochondrial electron transport complex IV, an essential component of the mitochondrial respiratory chain [58]. In lung cancer and melanoma, although COX10 deficiency reduces tumour neovascularization and slows tumour growth, it likewise leads to an increase in the area of avascular necrosis and promote tumour metastasis [59]. In addition, COX10-deficient cells upregulate glycolysis, which is the backbone of tumour cell metabolism [60, 61]. Thus, COX10 deficiency does more harm than good during tumour progression. In our study COX10 was significantly lower-expressed in the ESCC high-risk group in our study and was involved in the outcome of ESCC, which is consistent with this view. In addition, our *in vitro* experiments did find that in overexpression of COX10 could significantly inhibit the proliferation, migration and invasion ability of ESCC cells. Interestingly, previous reports have shown that multiple miRNAs can regulate the COX10 expression [58, 62]. In our interaction network analysis, COX10 similarly interacts with multiple miRNAs, suggesting that the expression of key genes for mitochondrial metabolism is multiply regulated in ESCC and cannot be simply generalized. Meanwhile, the crosstalk between these molecules also provides important clues for subsequent studies. In summary, our analysis indicated that key genes in the MMRG signature play an important role in the prognosis of ESCC patients, although previous studies have rarely reported their role in ESCC.

Certainly, there are some limitations to our study. First, the combined dataset did not contain prognostic information; thus, external validation of the clinical prediction model was not possible. Second, this study utilized public datasets for analysis, so there may be some inevitable selection biases. Otherwise, how these MMRGs affect the pathways and mechanisms of ESCC biogenesis needs to be explored through in-depth experiments. Finally, as limited by the original data, this study did not fully consider the impact of the location of oesophageal cancer and treatment methods, but the prediction rate of this study was above 90%; thus, this model is still very valuable.

## CONCLUSIONS

In conclusion, we investigated dysregulated mitochondrial metabolism-associated pathways in ESCC, and a novel 6 MMRGs signature in ESCC patients was developed that could accurately predict prognosis outcomes. This study might provide novel insights into predicting clinical outcomes of ESCC patients.

## Supplementary Material

Supplementary Figures

Supplementary Tables 1 and 2

Supplementary Table 3

Supplementary Tables 4 and 5

Supplementary Table 6

Supplementary Tables 7-9
