# Supplementary material for: A novel mitochondrial metabolism-related gene signature for predicting the prognosis of oesophageal squamous cell carcinoma
Source: Aging (Albany NY). 2024 Jun 5;16(11):9649–79. doi: 10.18632/aging.205892 (PMC11210263; doi:10.18632/aging.205892)
Supplement: Supplementary Figures [file aging-16-205892-s001.pdf]

SUPPLEMENTARY FIGURES

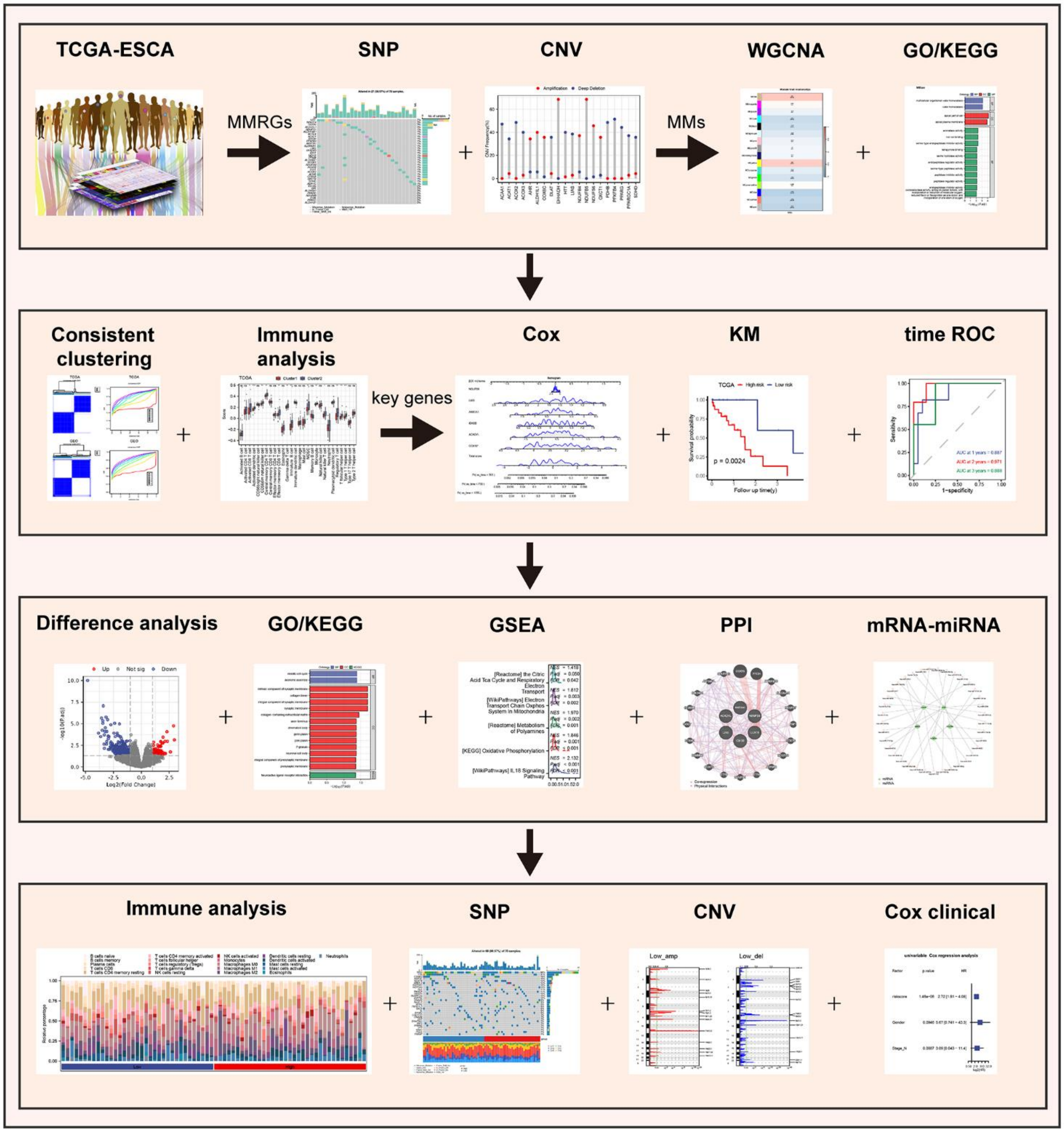

Supplementary Figure 1. Technology roadmap.

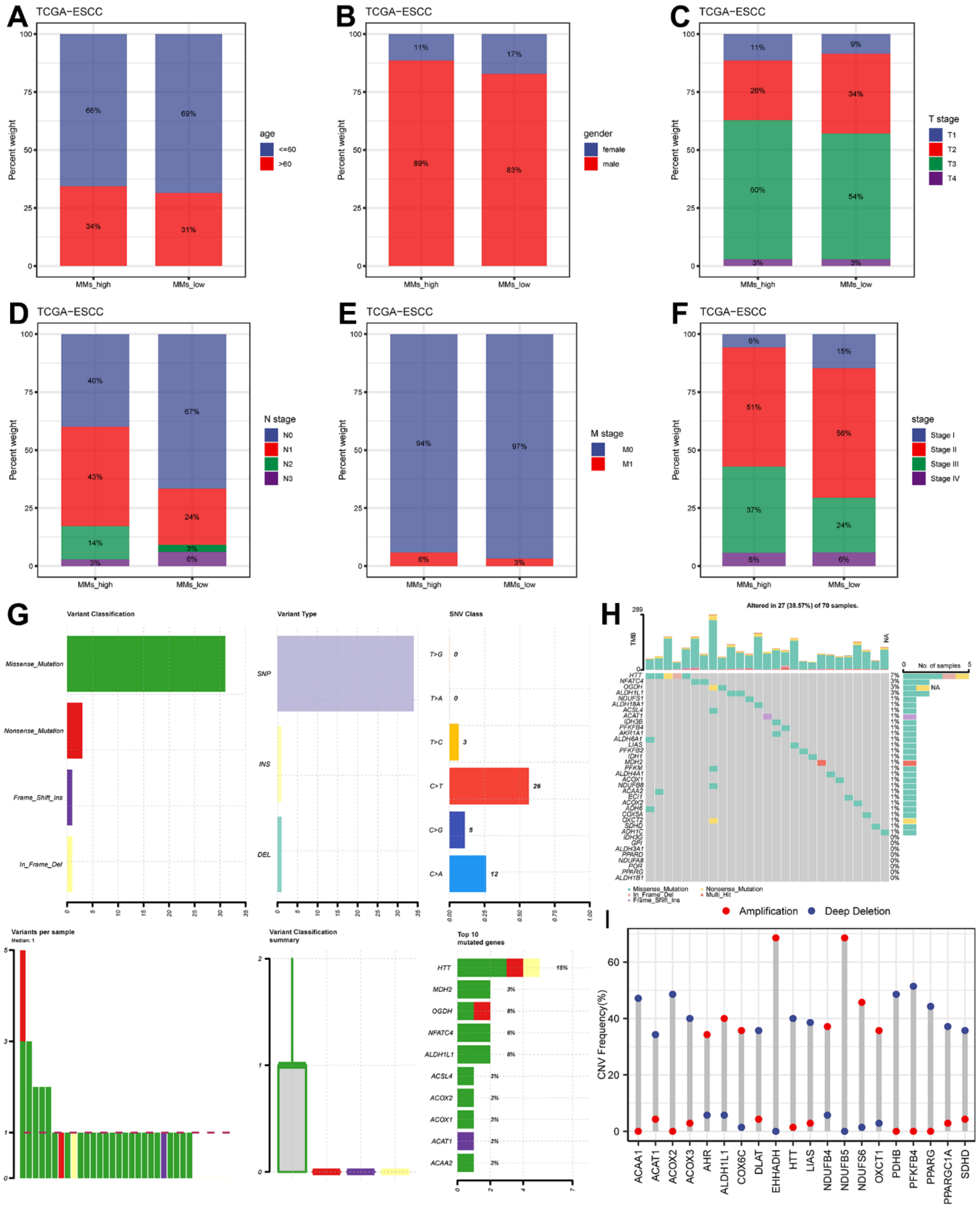

Supplementary Figure 2. Clinical correlation analysis of mitochondrial energy metabolism score and SNP/CNV analysis of MMRGs. (**A**–**F**) Clinical correlation analysis of mitochondrial energy metabolism score with Age (**A**), Gender (**B**), T stage (**C**), N stage (**D**), M stage (**E**), and Stage (**F**). (**G**) Overall SNP analysis of MMRGs in TCGA-ESCC. (**H**) Mutation waterfall plot of MMRGs in TCGA -ESCC. (**I**) CNV analysis of MMRGs in TCGA-ESCC. MMRGs, Mitochondrial metabolism-related genes. SNP, single nucleotide polymorphism. CNV, Copy Number Variation.

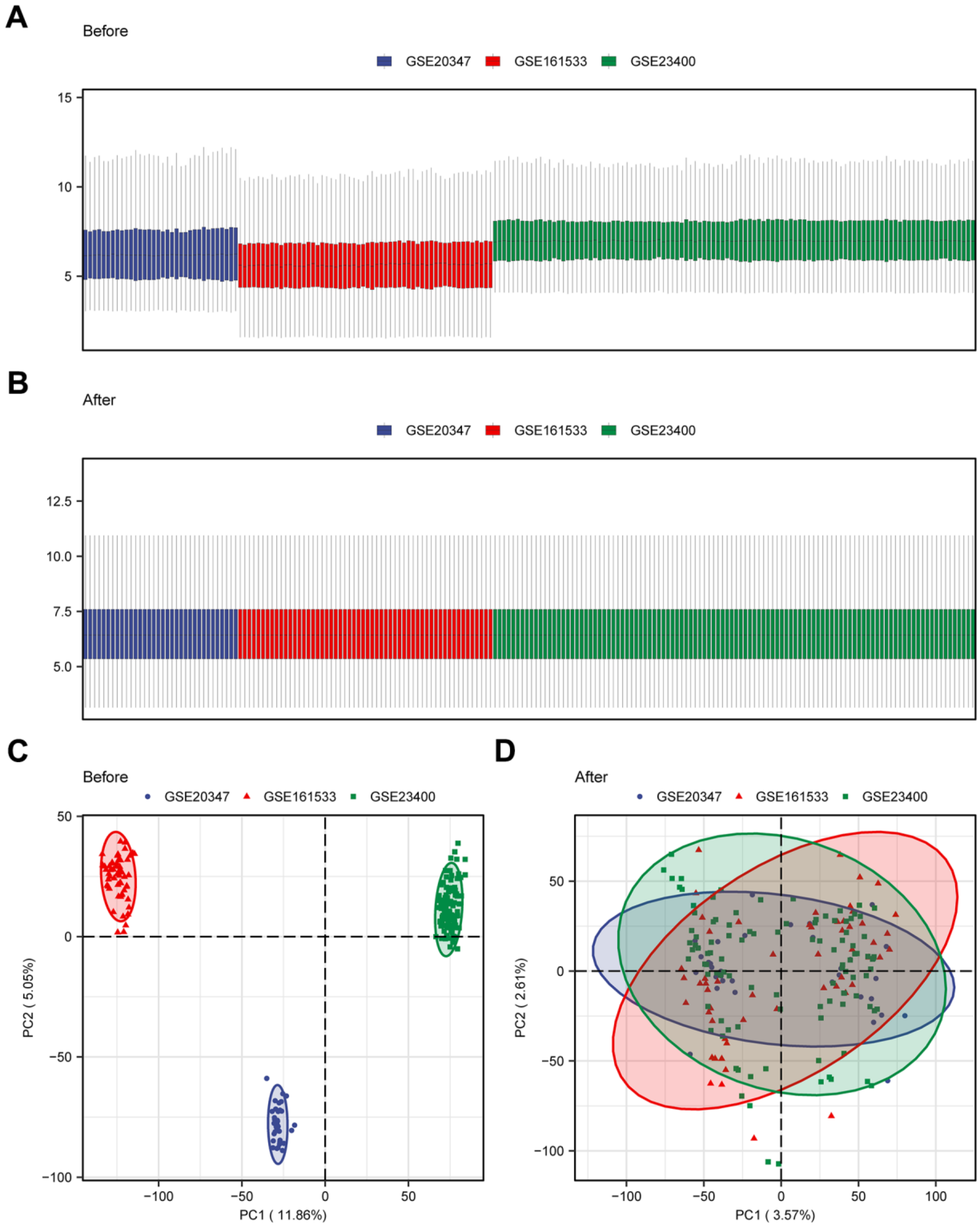

Supplementary Figure 3. Verify set collection and calibrate. (**A**) The boxplot plot of the merged dataset before removing the batch. (**B**) The boxplot plot of the merged dataset after removing batches. (**C**) Merge the PCA plot of the dataset before removing the batch. (**D**) PCA plot after merging the dataset and removing batches. PCA: Principal Component Analysis.

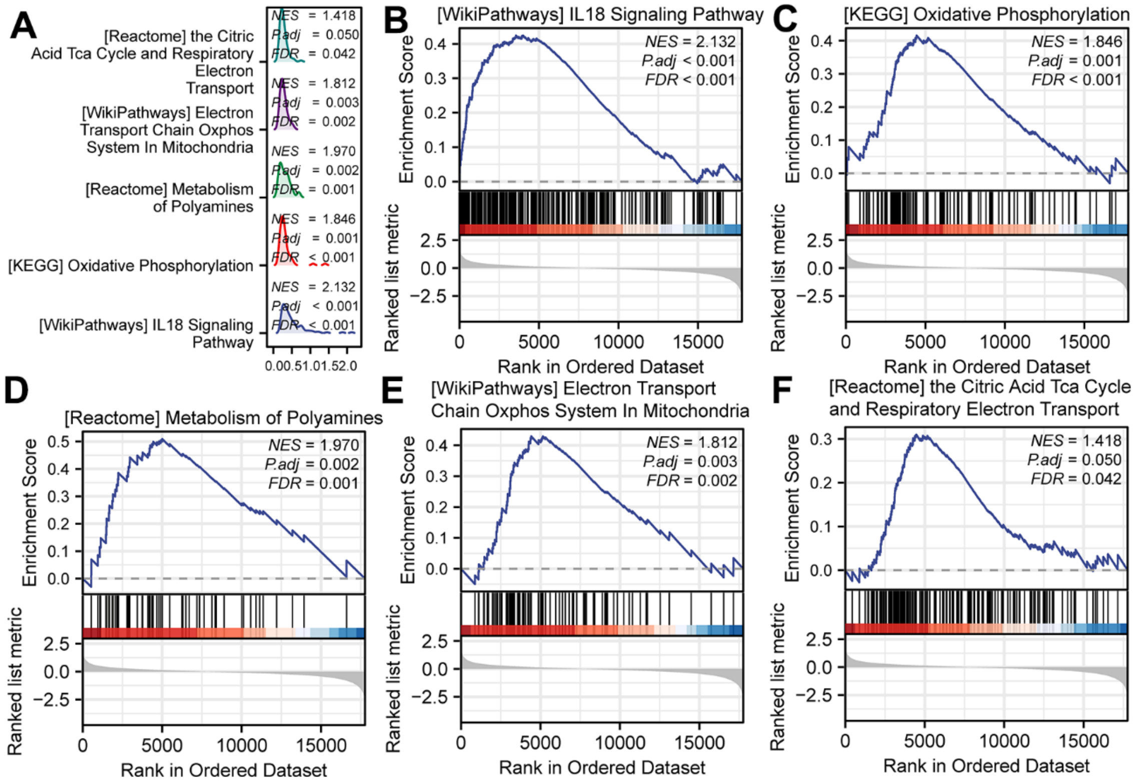

Supplementary Figure 4. The GSEA of the TCGA-ESCC dataset. (**A**) Volcano plot of the GSEA enrichment analysis of the TCGA-ESCC dataset. (**B**–**F**) Pathway maps of the IL 18 Signaling Pathway (**B**), KEGG Oxidative phosphorylation (**C**), Metabolism of polyamines (**D**), Electron transport chain OXPHOS system in mitochondria (**E**), and Electron Transport (**F**) pathways.

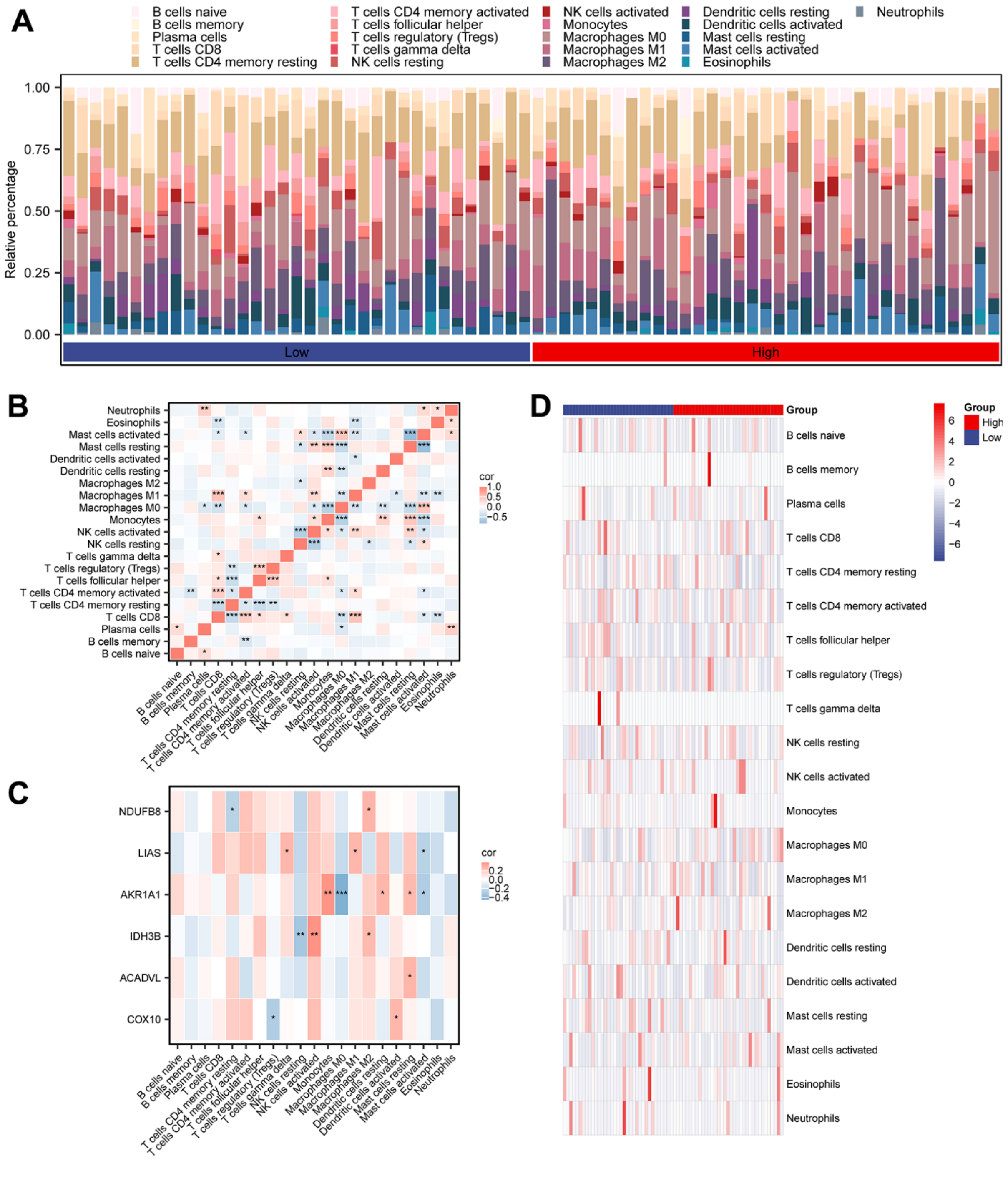

Supplementary Figure 5. Immune infiltration analysis of the TCGA-ESCC. (**A**) Stacked bar chart of immune cell infiltration in the High and Low groups of the TCGA-ESCC. (**B**) Correlation heatmap of immune cells. (**C**) Correlation heatmap of immune cells and key genes. (**D**) Heatmap of immune cell infiltration in the TCGA-ESCC.

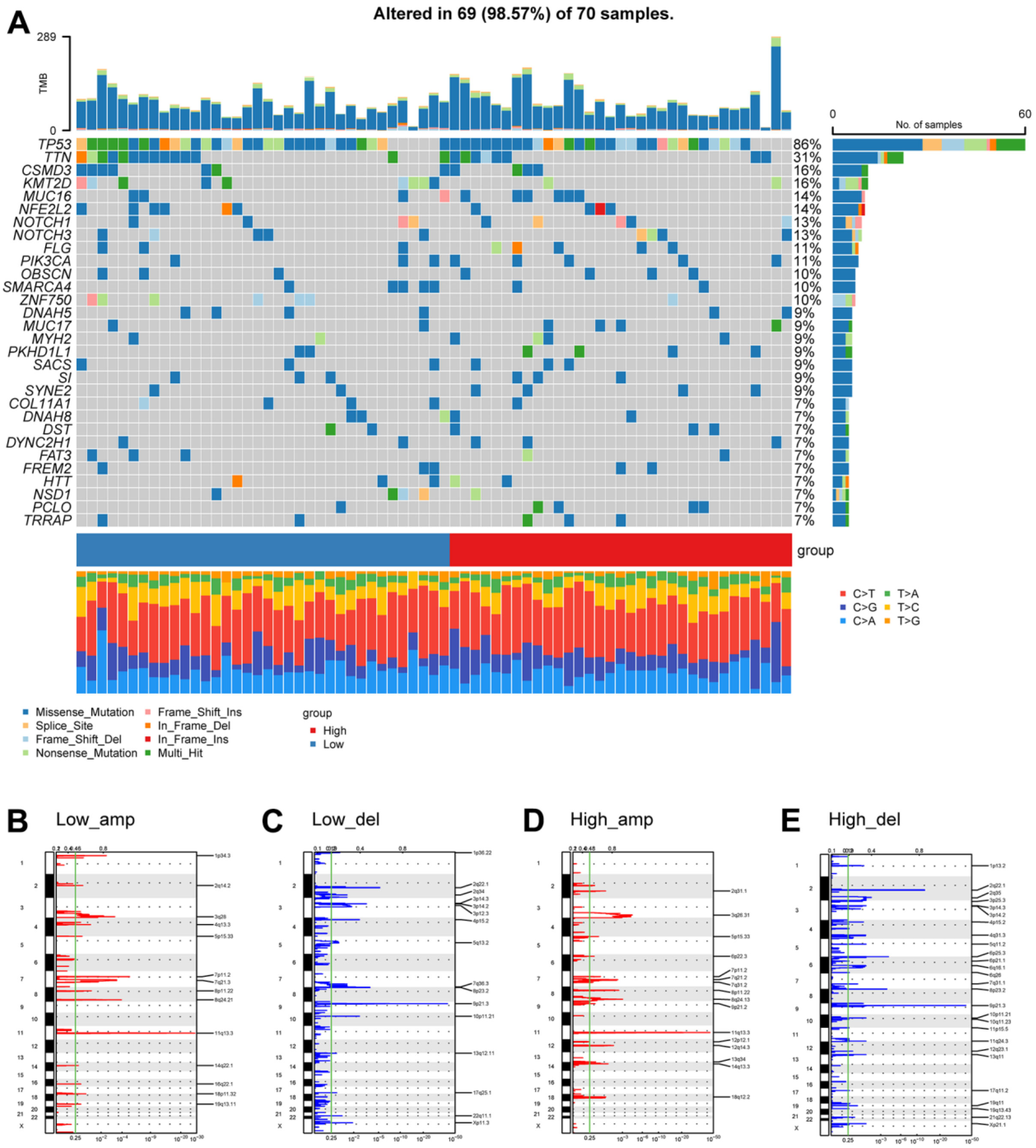

Supplementary Figure 6. SNP and CNV analysis of high-risk and low-risk groups in the Cox model. (**A**) Waterfall plot of mutation analysis for high- and low-risk groups in the Cox model. (**B**, **C**) GISTIC analysis of the low-risk group in the TCGA-ESCC dataset. Red indicates CNV amplification (**B**), while blue denotes CNV deletion (**C**). (**D**, **E**) GISTIC analysis of the high-risk group in the TCGA-ESCC dataset. Red represents CNV amplification (**D**), while blue represents CNV deletion (**E**).

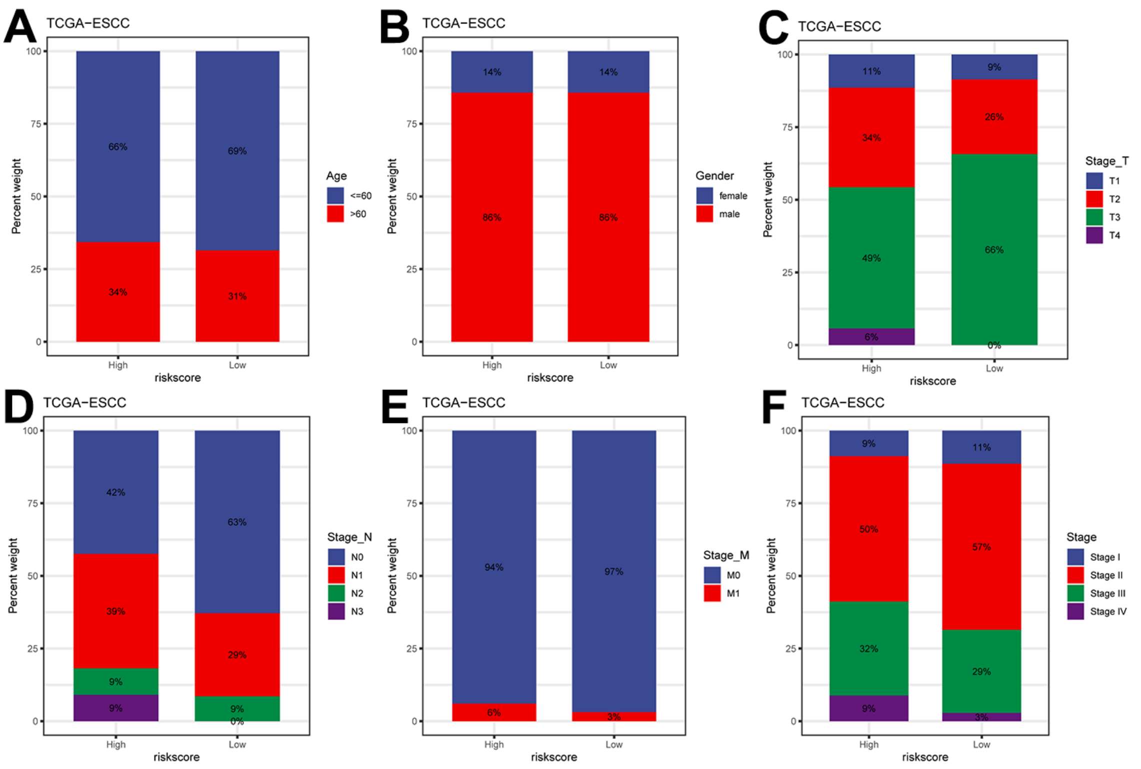

Supplementary Figure 7. Clinical prediction model based on risk score in the TCGA-ESCC dataset. (**A**–**F**) Clinical correlation analysis of mitochondrial energy metabolism score with Age (**A**), Gender (**B**), T stage (**C**), N stage (**D**), M stage (**E**), and Stage (**F**).

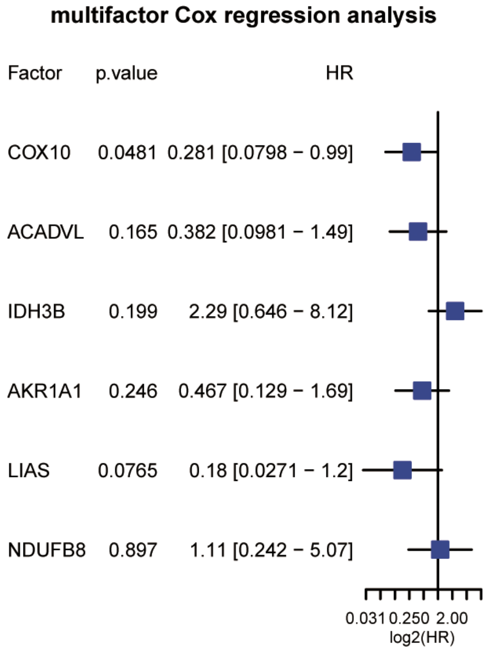

Supplementary Figure 8. Risk factor map of MMRGs prognostic model.

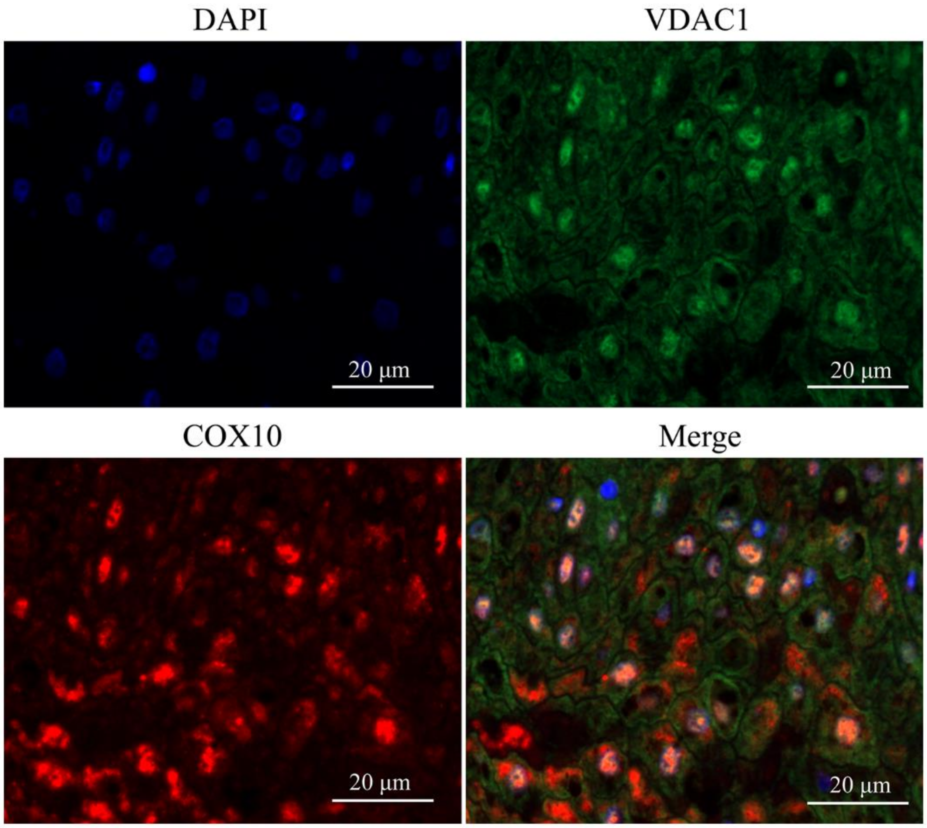

Supplementary Figure 9. Immunofluorescence quantitative expression of COX10 in normal oesophageal tissues.
