# Supplementary material for: A novel mitochondrial metabolism-related gene signature for predicting the prognosis of oesophageal squamous cell carcinoma
Source: Aging (Albany NY). 2024 Jun 5;16(11):9649–79. doi: 10.18632/aging.205892 (PMC11210263; doi:10.18632/aging.205892)
Supplement: Supplementary Tables 1 and 2 [file aging-16-205892-s002.pdf]

SUPPLEMENTARY TABLES

Supplementary Table 1. List of MMRGs.

NDUFAB1	ALDH3A1	COX7B	NDUFB8
ALDH3B1	NDUFC1	ACLY	COX11
COX10	PPARGC1A	NDUFA2	IDH3A
MDH1	CPT1A	PPARG	ACAA2
COX15	PDHX	ALDH3B2	NDUFV1
NDUFS1	ALDH2	COX5B	ECI1
ALDH18A1	GAPDH	NDUFB5	PDHB
ACAA1	PPARD	ALDH1B1	ACOX2
CS	ALDH5A1	HADHB	UQCRFS1
NDUFB4	EHHADH	IDH1	PFKFB3
PFKP	PFKFB4	PPA2	NDUFA3
IDH3G	NDUFS7	HADH	BPGM
ACSL4	ACADL	NDUFA9	ADH6
ALDH3A2	PDC	ETFA	PC
ACADVL	ACADM	PFKL	PAAF1
SDHA	SDHB	ALDH9A1	NDUFV2
ACAT1	HMGCL	SDHC	COX5A
ATP12A	AKR1A1	PKLR	CYC1
OXCT1	ALDH8A1	ALDH1L1	PPA1
HADHA	NDUFB3	NDUFS6	NDUFB1
ACOX3	NDUFA8	MDH2	ALDH1A3
NDUFB2	DLST	NDUFB11	NDUFA6
DLD	ALDH6A1	DLAT	ATP4B
NDUFB7	ACAT2	ACSL1	NDUFA13
ACO2	LIAS	PFKM	PPARA
NFATC4	ACO1	CYP2U1	CYP4A11
IDH3B	ACADS	CPT2	ADH1A
ACSBG1	PFKFB2	PFKFB1	NDUFA4
GPI	ACSL3	NDUFS2	ACADSB
GCDH	NDUFA1	ADPGK	ADH1B
ATP4A	COX7C	ALDH4A1	ACSL5
GAPDHS	ECHS1	ACOX1	HTT
OGDH	POR	NDUFS4	ECI2
AHR	PPAT	ACSL6	OXCT2
GCK	NDUFA5	ALDH7A1	SDHD
MINPP1	ALDH1A2	COX6C	NDUFS3
LHPP	ACSBG2	ALDH1A1	ADH1C
C1QBP	NDUFA10	NDUFB6	NDUFA7

Supplementary Table 2. The details of antibodies.

Name	Catalog No.	Company
ACADVL	14527-1-AP	Proteintech
COX10	10611-2-AP	Proteintech
IDH3B	K006914P	Solarbio
AKR1A1	15054-1-AP	Proteintech
LIAS	11577-1-AP	Proteintech
NDUFB8	14794-1-AP	Proteintech
VDAV1	GB111939	Servicebio
