# Supplementary material for: A novel mitochondrial metabolism-related gene signature for predicting the prognosis of oesophageal squamous cell carcinoma
Source: Aging (Albany NY). 2024 Jun 5;16(11):9649–79. doi: 10.18632/aging.205892 (PMC11210263; doi:10.18632/aging.205892)
Supplement: Supplementary Table 3 [file aging-16-205892-s003.docx]

**Supplementary Table 3. List of MEtan and MEblue related genes.**

| **MEtan** | ***MEblue*** |  |  |  |  |  |
| --- | --- | --- | --- | --- | --- | --- |
| SPRR3 | *CST1* | *CSF2* | *ITGAV* | *CNTNAP1* | *SNX10* | *PCDHB15* |
| KRT13 | *S100P* | *PDGFRB* | *GJC1* | *AXL* | *DCLK2* | *ADAMTS10* |
| CEACAM5 | *CSPG4* | *IRS1* | *ESM1* | *KLF10* | *MACF1* | *RAB30* |
| TMPRSS11E | *SFRP4* | *CPE* | *OMD* | *SLC6A2* | *PKIG* | *MAN2B1* |
| KRT4 | *COL10A1* | *CYP7B1* | *TNFRSF12A* | *FXYD6* | *FKBP9* | *PDE4D* |
| TMPRSS11A | *COL11A1* | *CORO6* | *ITGA1* | *GLIS2* | *FKBP14* | *TMEM231* |
| LYPD2 | *FN1* | *DDR2* | *TTYH2* | *LMCD1* | *ADGRB2* | *RAB8B* |
| SERPINB4 | *SPP1* | *FMOD* | *IGFBP7* | *PLXND1* | *MFGE8* | *ENPP1* |
| CXCL17 | *POSTN* | *PLAU* | *RTN4RL2* | *ARHGEF40* | *TCEAL3* | *EIF5A2* |
| DAPL1 | *COL5A2* | *MSRB3* | *SYT11* | *CLIP2* | *KCTD17* | *NPTX2* |
| LCN2 | *MMP1* | *CCDC80* | *KLF9* | *MRAS* | *SKIL* | *RAMP2* |
| SLURP1 | *SERPINE1* | *TNFRSF19* | *HAAO* | *VGLL3* | *TDO2* | *TBXA2R* |
| SERPINB3 | *POPDC3* | *FHOD3* | *SMIM22* | *CHN1* | *HHIPL1* | *RGL1* |
| CEACAM6 | *INHBA* | *OLFML3* | *RCAN2* | *SLC4A3* | *FHL3* |  |
| NCCRP1 | *MMP3* | *COLEC12* | *CPA3* | *PAPSS2* | *KIAA1755* |  |
| CLCA4 | *THBS2* | *NR4A3* | *ALDH1B1* | *LHFPL2* | *PI15* |  |
| CRNN | *LAMC2* | *ATP8B2* | *MEIS3* | *STARD13* | *PPP1R18* |  |
| EPHX3 | *MMP13* | *CD248* | *ADGRA2* | *DTX3* | *EFHD1* |  |
| SERPINB13 | *TSPAN18* | *RARRES2* | *TGFB1I1* | *CEMIP2* | *TTYH3* |  |
| TCN1 | *COL3A1* | *CCL11* | *GNB4* | *ARHGEF25* | *SPRY4* |  |
| CYSRT1 | *COL1A1* | *C1QTNF6* | *MFAP2* | *ZNF608* | *TBC1D1* |  |
| SPINK5 | *MSC* | *SHISA4* | *RETREG1* | *TRIO* | *ADAMTS9* |  |
| KRT78 | *SFRP2* | *MICAL2* | *OLFML1* | *FOXF2* | *PRR16* |  |
| MAL | *LRRC15* | *IL6* | *SPOCD1* | *DSE* | *BICDL1* |  |
| PCP4L1 | *MMP11* | *MAP9* | *SELENOM* | *SGPP1* | *CTF1* |  |
| B3GNT3 | *TNC* | *FLNA* | *FGFR1* | *ITPKB* | *ZYX* |  |
| TMEM45B | *VCAN* | *DEPP1* | *HEYL* | *PCDH18* | *RTL5* |  |
| IL36A | *COL6A3* | *BHLHE41* | *TMEM255A* | *ID3* | *NEDD4* |  |
| TMPRSS11F | *COL5A1* | *LTBP2* | *GPR155* | *WHRN* | *IKBIP* |  |
| FUT3 | *COL1A2* | *GRP* | *COPZ2* | *BMP8A* | *ANXA5* |  |
| SCNN1B | *EDIL3* | *BICC1* | *L1CAM* | *CEP112* | *DPYSL2* |  |
| MUC15 | *COL12A1* | *THY1* | *CD200* | *PKD2* | *SESTD1* |  |
| CLIC3 | *CTSK* | *NID1* | *RGS4* | *MPP2* | *PTGFRN* |  |
| POF1B | *MAP1B* | *NTM* | *CYS1* | *TMEM204* | *PAM* |  |
| ALOX15B | *CAV1* | *C11orf96* | *MET* | *RNF144A* | *SPSB1* |  |
| VSIG10L | *MMP2* | *TMEM119* | *SNAI1* | *CYYR1* | *NECAB1* |  |
| PRSS3 | *GAS1* | *ADAMTS1* | *FBLN5* | *MORN4* | *KLHL5* |  |
| MAB21L4 | *LUM* | *SYNPO* | *IL31RA* | *BNC2* | *EEPD1* |  |
| CSTA | *ISLR* | *GASK1B* | *ITGA4* | *LTBP3* | *BMF* |  |
| TJP3 | *COL6A1* | *PLXDC2* | *TWSG1* | *SOX18* | *ORAI2* |  |
| SUSD4 | *ADAMTS2* | *HMCN1* | *SMARCD3* | *COL7A1* | *JAZF1* |  |
| CYP2C18 | *PDPN* | *EMP3* | *CAVIN3* | *ANO6* | *LGR5* |  |
| LRRC4 | *SULF1* | *TGFBR2* | *PRKG1* | *HAS2* | *USB1* |  |
| PPP1R3C | *CACNA2D1* | *RHOBTB3* | *APBB1* | *SALL4* | *VPS13C* |  |
| GGT6 | *SRPX* | *WNT2* | *TSHZ3* | *ADAMTS5* | *ODF3L1* |  |
| CEACAM1 | *GPC6* | *STC1* | *PRKCA* | *PPFIBP1* | *NAP1L3* |  |
| SCNN1A | *GREM1* | *DKK3* | *CHST11* | *TNIK* | *GADD45A* |  |
| FAM83E | *TGFBI* | *SLIT2* | *MXRA7* | *GPRC5B* | *STARD9* |  |
| FOXA1 | *CCDC8* | *NUAK1* | *SSPN* | *NFATC4* | *SPATA20* |  |
| BNIPL | *ADAMTS15* | *CCDC3* | *PMP22* | *CHPF* | *PALLD* |  |
| DHRS9 | *APCDD1L* | *LZTS1* | *LGI2* | *PGM2L1* | *MAP3K20* |  |
| PSCA | *PRRX1* | *EBF4* | *SNAI2* | *CALCRL* | *LZTS2* |  |
| SLC6A14 | *FAP* | *TUBA1A* | *TMEM200A* | *MME* | *COMTD1* |  |
| LRG1 | *ROR2* | *DACT1* | *KCNE4* | *CYP2U1* | *NCALD* |  |
| ACP3 | *PALM* | *TRPC1* | *ZEB1* | *ANGPT1* | *SYTL2* |  |
| PRSS27 | *ASPN* | *FILIP1L* | *ENPEP* | *MMP14* | *BTBD19* |  |
| SPINK7 | *SPOCK1* | *FBXL7* | *COL15A1* | *PLPP3* | *IFFO1* |  |
| PAX9 | *COL8A1* | *NDN* | *FMO1* | *SMIM10* | *ZEB2* |  |
| TTC9 | *MXRA5* | *TMEM47* | *NEK6* | *PCDH17* | *FGD5* |  |
| FMO2 | *MYADM* | *LAMP5* | *MEDAG* | *CSGALNACT1* | *LRCH2* |  |
| RRAD | *FBN1* | *FSTL1* | *P4HA2* | *CAP2* | *TMEM263* |  |
| MUC20 | *SEMA3C* | *SCARF2* | *GJA5* | *FHL2* | *NHSL2* |  |
| HS3ST6 | *F2RL2* | *CLIP3* | *CDYL2* | *SYDE1* | *RFLNA* |  |
| MUC4 | *BASP1* | *SPON1* | *PTGER4* | *LHFPL6* | *RTN2* |  |
| OTOP3 | *MFAP4* | *FIBIN* | *PRKD1* | *ST6GALNAC5* | *ULK2* |  |
| ATP10B | *BGN* | *APLN* | *ADARB1* | *SH3PXD2B* | *BPGM* |  |
| ST6GALNAC1 | *CTHRC1* | *CDK14* | *PODNL1* | *PAPLN* | *FOXS1* |  |
| UPK3B | *BVES* | *HTRA1* | *RAB31* | *PLPPR2* | *CCDC102A* |  |
| RNF225 | *CX3CL1* | *VIM* | *MATN3* | *GPER1* | *PTGER3* |  |
| ELF3 | *PEG10* | *PDGFRL* | *CAVIN1* | *DYNC2H1* | *WLS* |  |
| EHF | *GXYLT2* | *PALM2AKAP2* | *RAB23* | *ADRA2A* | *RIN3* |  |
| ARHGAP40 | *MFAP5* | *MAPK8IP1* | *TGFB3* | *MICALL2* | *SLC2A13* |  |
| SOWAHB | *HTRA3* | *MAP1A* | *CCDC113* | *GLS* | *ANTXR2* |  |
| MANSC1 | *VCAM1* | *ELMO1* | *HOXB2* | *COX7A1* | *RIPOR3* |  |
| DEGS2 | *MN1* | *SLC2A3* | *CRISPLD2* | *REEP2* | *TCEAL7* |  |
| DUOX2 | *FNDC1* | *ACTN1* | *NEDD9* | *PDLIM7* | *CALHM5* |  |
| RASAL1 | *CCN2* | *PODN* | *FMNL2* | *AXIN2* | *APOLD1* |  |
| LIPH | *COL5A3* | *ANOS1* | *STON1* | *MSN* | *CHST6* |  |
| SH3BGRL2 | *LOXL2* | *ZNF521* | *PLPPR4* | *SHC2* | *ITGBL1* |  |
| TMPRSS2 | *GPR68* | *DOCK11* | *SH3RF3* | *FBLIM1* | *SHROOM4* |  |
| HOPX | *EGR2* | *GUCY1A1* | *CPQ* | *PRKACB* | *BCL6B* |  |
| FA2H | *ZNF469* | *SGCD* | *GLIPR1* | *CFH* | *CSGALNACT2* |  |
| DUOXA2 | *PXDN* | *TOX2* | *MCAM* | *ARHGAP31* | *ITGB3* |  |
| PCDH1 | *CDH11* | *TGFB2* | *DNAJB4* | *RBMS3* | *RNF122* |  |
| USH1G | *FERMT2* | *CRMP1* | *CAV2* | *NOX4* | *HOXB5* |  |
| HPGD | *ITGA3* | *LIFR* | *HOXB3* | *GALC* | *RAB32* |  |
| PADI1 | *OLFM2* | *MXRA8* | *SLC39A14* | *RUSC2* | *MBNL2* |  |
| PRR15L | *ADAM12* | *ANTXR1* | *LRIG1* | *LBH* | *SCT* |  |
| ABLIM1 | *CCN4* | *COMP* | *PRR5L* | *TNFSF4* | *HRH1* |  |
| CYP4F12 | *OLFML2B* | *SUGCT* | *FGF1* | *PDK3* | *ANKH* |  |
| IL27RA | *SDC2* | *S1PR3* | *PLEKHA4* | *FOXD3* | *DYSF* |  |
| TM7SF2 | *CHI3L1* | *NEXN* | *KCNMB4* | *EGFL6* | *AL121753.1* |  |
| MAB21L3 | *GFPT2* | *ADAMTS14* | *SLC2A10* | *GALNT15* | *TAMALIN* |  |
| BICDL2 | *SPARC* | *CALD1* | *ADAMTS7* | *CHSY1* | *KCNIP3* |  |
| LNX1 | *DUSP6* | *LAMA4* | *MTCL1* | *APP* | *NOTCH4* |  |
| SNX31 | *NID2* | *GUCY1B1* | *ITGB1* | *KDR* | *PHYH* |  |
| CLDN4 | *ADAMTS12* | *CMTM3* | *ETV1* | *IL1R1* | *GJA4* |  |
| NDRG2 | *ANGPTL2* | *SPEG* | *ANXA6* | *FAM168A* | *WWC2* |  |
| IKZF2 | *COL6A2* | *SSC5D* | *PLXNC1* | *ARHGEF17* | *NPTN* |  |
| TP53INP2 | *AEBP1* | *CERCAM* | *FKBP7* | *CDH2* | *MAPRE2* |  |
| ATP13A4 | *PCDH7* | *DAB2* | *C2CD4B* | *BMERB1* | *TMEM25* |  |
| SPNS2 | *LTBP1* | *NRP2* | *COL4A2* | *TIE1* | *LRRN4CL* |  |
| GCNT3 | *FSTL3* | *CYBRD1* | *IQCN* | *SGCE* | *RCAN1* |  |
| MYO5B | *EMILIN1* | *GLI3* | *ZNF71* | *TRO* | *FLT1* |  |
| BSPRY | *TENM3* | *KANK2* | *IFITM2* | *C1QTNF3* | *ARL15* |  |
| ANKRD35 | *CPXM1* | *XYLT1* | *MEGF6* | *KCTD12* | *LIX1L* |  |
| DBNDD1 | *COL16A1* | *CILP2* | *RHOU* | *ARID5B* | *SERP2* |  |
| EMP1 | *GPX7* | *TWIST1* | *TIMP1* | *ZCCHC24* | *GLRB* |  |
| SPAG17 | *SULF2* | *NPR2* | *CDC42EP3* | *RFLNB* | *TSPAN9* |  |
| STX19 | *LOX* | *GLT8D2* | *VSTM4* | *MPDZ* | *HELB* |  |
| TTC39A | *EPDR1* | *ISM1* | *NRP1* | *SCUBE2* | *APBB2* |  |
| C15orf48 | *FKBP10* | *LIMA1* | *PDZD4* | *HSPG2* | *TSPAN11* |  |
| EPB41L1 | *PDLIM3* | *FBLN1* | *KIRREL1* | *OSBPL7* | *ADGRF5* |  |
| ENDOU | *SEMA6D* | *MRGPRF* | *SOCS2* | *PBX3* | *HSD11B1* |  |
| RNF208 | *NNMT* | *UCN2* | *GYG2* | *COL24A1* | *DIP2C* |  |
| MACC1 | *COL27A1* | *HEG1* | *ARHGAP28* | *ZNF221* | *PIP4P2* |  |
| LDHD | *RASD2* | *CXCL12* | *DUSP10* | *FAM110B* | *BICD1* |  |
| FAM3D | *ITGA11* | *CHST10* | *KANK4* | *P3H4* | *FNIP2* |  |
| BLNK | *ENO2* | *C8orf88* | *ENC1* | *DCLK1* | *LURAP1* |  |
| SERPINB11 | *SPON2* | *COL4A1* | *BMP1* | *VWF* | *ITGB5* |  |
| TPRG1 | *ZNF853* | *ZFHX4* | *ITM2C* | *PNMA1* | *BACH2* |  |
| FUT6 | *BCAT1* | *JCAD* | *EVC* | *RECK* | *ZBED1* |  |
| BBOX1 | *ITGA5* | *THBS1* | *DPP4* | *GPSM1* | *ZNF319* |  |
| OCLN | *ALDH1L2* | *CLEC11A* | *SMIM3* | *STARD3NL* | *SCG2* |  |
| RASGRP1 | *TSPAN2* | *LRP1* | *UACA* | *CYRIA* | *VASH1* |  |
| CGN | *FBLN2* | *ARHGAP29* | *TBX5* | *MAML2* | *DYRK2* |  |
| SYTL5 | *TWIST2* | *RAB3IL1* | *HDAC9* | *KCNJ8* | *SEMA3B* |  |
| LMO7 | *LOXL1* | *PRRX2* | *SCG5* | *CTSO* | *GUCY1A2* |  |
| RAB11FIP1 | *DCN* | *TAFA5* | *GLIS3* | *GLIS1* | *PLEKHG1* |  |
| TRNP1 | *C12orf75* | *KIFC3* | *FEZ1* | *ST3GAL2* | *SPHK1* |  |
| CAPN5 | *PRSS23* | *EFEMP2* | *PTPRM* | *IGFBP4* | *B3GNT9* |  |
| ATP6V1C2 | *GEM* | *ADAM19* | *DLC1* | *PDGFRA* | *POGLUT2* |  |
| PLEKHA7 | *RCN3* | *ECM2* | *SGCB* | *INSR* | *ZNF267* |  |
| SLC7A4 | *LRRC17* | *GAS6* | *NES* | *ARL4C* | *APLP1* |  |
| SMIM5 | *AKT3* | *COL14A1* | *CAND2* | *TCF4* | *ZNF792* |  |
| CAPN14 | *DPYSL3* | *AMIGO2* | *GATA6* | *PTP4A3* | *PLAUR* |  |
| KRT24 | *SERPINF1* | *PDGFC* | *BTNL9* | *TMEM121* | *CTIF* |  |
| TMEM125 | *NR4A1* | *RASSF8* | *CTSF* | *SLC12A4* | *GALNT10* |  |
| CDH16 | *LGALS1* | *NLGN2* | *PLEKHH2* | *MMD* | *TM4SF18* |  |
| CYP4B1 | *C1QTNF1* | *LAMB1* | *FLRT2* | *CNRIP1* | *SYNC* |  |
| CEACAM7 | *PGF* | *KIF26B* | *FMNL3* | *SMTN* | *CALU* |  |
| CXCR2 | *PMEPA1* | *RHOBTB1* | *CRISPLD1* | *TLN2* | *NR2F2* |  |
| ANXA9 | *EDNRA* | *WNT5B* | *EML1* | *SPECC1* | *TTC28* |  |
| LEXM | *CCN1* | *IGFBPL1* | *P4HA1* | *SNCA* | *PCDH12* |  |
| TLR5 | *KIAA1549L* | *TPM1* | *KDELR3* | *P3H1* | *EOGT* |  |
| C15orf62 | *SEMA3A* | *TTLL7* | *C20orf96* | *FUT8* | *CLEC5A* |  |
| EPB41L4A | *EVA1A* | *MRC2* | *LAMA2* | *ZFPM2* | *LPCAT2* |  |
| ADGRF1 | *CST2* | *RUNX2* | *F2R* | *GPM6B* | *SPATA6* |  |
| TMPRSS11B | *SHISAL1* | *SYNDIG1* | *KCND2* | *CNN3* | *UTRN* |  |
| FAM3B | *TNFAIP6* | *DCHS1* | *GPX8* | *PRICKLE1* | *CFL2* |  |
| SLURP2 | *CDH13* | *F13A1* | *PHLDB1* | *PYROXD2* | *ZNF358* |  |
| RIPK4 | *DCBLD1* | *SLC16A2* | *MARVELD1* | *ZNF423* | *BRSK1* |  |
| SH2D2A | *RGS16* | *MSX2* | *TACC1* | *BEND6* | *SAMD10* |  |
| CES2 | *TIMP3* | *FGF2* | *CHURC1* | *CD99L2* | *CD101* |  |
| SASH1 | *MAN1A1* | *HOXB4* | *PTPRG* | *PXN* | *PROC* |  |
| CRYBG1 | *TIMP2* | *APLNR* | *LAMB2* | *SEC24D* | *IGDCC4* |  |
| N4BP3 | *AKAP12* | *APBA2* | *PDE3A* | *CCDC74A* | *CSDC2* |  |
| TP53I3 | *PKDCC* | *CHSY3* | *ACOT1* | *CD302* | *LDLRAD4* |  |
| SLC16A6 | *PCOLCE* | *ARMCX2* | *SAMD4A* | *MAGEH1* | *PEAR1* |  |
| AGFG2 | *P3H3* | *FOXF1* | *LRRC32* | *ZNF25* | *ARMC9* |  |
| SERPINB12 | *C1QL1* | *LRP3* | *PDGFB* | *PLXDC1* | *ZNF699* |  |
| MUC21 | *STMN3* | *TPM2* | *SLC22A17* | *TES* | *MAP4* |  |
| CYP3A5 | *COL8A2* | *HEPH* | *SERPINH1* | *MITF* | *SLC22A4* |  |
| PDCD4 | *CLMP* | *GGT5* | *CRACD* | *SVEP1* | *NTN4* |  |
| GCOM1 | *SLIT3* | *ADAMTS4* | *MMP19* | *LSAMP* | *RHOJ* |  |
| FABP12 | *PLPP4* | *LIMCH1* | *SUSD5* | *JAM3* | *MMP16* |  |
| ARHGEF10L | *CREB3L1* | *IL11* | *HEY2* | *ACO1* | *PLEKHO1* |  |
| NBEAL2 | *GNAZ* | *COL18A1* | *C3orf80* | *PIEZO1* | *CLIC4* |  |
| OTOP2 | *PLEKHG4B* | *PKN1* | *GADD45B* | *GNA12* | *EGFLAM* |  |
