# Supplementary material for: A novel mitochondrial metabolism-related gene signature for predicting the prognosis of oesophageal squamous cell carcinoma
Source: Aging (Albany NY). 2024 Jun 5;16(11):9649–79. doi: 10.18632/aging.205892 (PMC11210263; doi:10.18632/aging.205892)
Supplement: Supplementary Tables 4 and 5 [file aging-16-205892-s004.pdf]

Supplementary Table 4. GO and KEGG enrichment analysis results of MEtan related genes.

ONTOLOGY	ID	Description	GeneRatio	BgRatio	p-value	p.adjust	q-value
BP	GO:0050891	multicellular organismal water homeostasis	7/151	59/18800	4.51E-07	0.000673378	0.000648524
BP	GO:0030104	water homeostasis	7/151	66/18800	9.82E-07	0.000733374	0.000706306
CC	GO:0045177	apical part of cell	16/158	424/19594	3.47E-07	6.45E-05	6.06E-05
CC	GO:0016324	apical plasma membrane	14/158	358/19594	1.28E-06	0.000118886	0.000111687
MF	GO:0004867	serine-type endopeptidase inhibitor activity	7/154	98/18410	1.83E-05	0.004830767	0.004301305
MF	GO:0005506	iron ion binding	8/154	151/18410	4.04E-05	0.004830767	0.004301305
MF	GO:0070330	aromatase activity	4/154	25/18410	5.19E-05	0.004830767	0.004301305
MF	GO:0004866	endopeptidase inhibitor activity	8/154	180/18410	0.00013859	0.007255809	0.006460558
MF	GO:0061134	peptidase regulator activity	9/154	230/18410	0.000139868	0.007255809	0.006460558
MF	GO:0030414	peptidase inhibitor activity	8/154	187/18410	0.00018012	0.007255809	0.006460558
MF	GO:0008236	serine-type peptidase activity	8/154	191/18410	0.000208121	0.007255809	0.006460558
MF	GO:0061135	endopeptidase regulator activity	8/154	194/18410	0.000231388	0.007255809	0.006460558
MF	GO:0017171	serine hydrolase activity	8/154	195/18410	0.000239602	0.007255809	0.006460558
MF	GO:0046906	tetrapyrrole binding	7/154	149/18410	0.000260065	0.007255809	0.006460558
MF	GO:0016712	oxidoreductase activity, acting on paired donors, with incorporation or reduction of molecular oxygen, reduced flavin or flavoprotein as one donor, and incorporation of one atom of oxygen	4/154	40/18410	0.000340417	0.008634215	0.007687888

Supplementary Table 5. GO and KEGG enrichment analysis results of MEblue related genes.

ONTOLOGY	ID	Description	GeneRatio	BgRatio	p-value	p.adjust	q-value
BP	GO:0030198	extracellular matrix organization	108/815	307/18800	3.43E-69	1.20E-65	9.18E-66
BP	GO:0043062	extracellular structure organization	108/815	308/18800	5.09E-69	1.20E-65	9.18E-66
BP	GO:0045229	external encapsulating structure organization	108/815	310/18800	1.11E-68	1.74E-65	1.34E-65
BP	GO:0031589	cell-substrate adhesion	76/815	364/18800	4.09E-31	4.81E-28	3.69E-28
CC	GO:0062023	collagen-containing extracellular matrix	129/839	429/19594	7.25E-74	3.15E-71	2.54E-71
CC	GO:0005788	endoplasmic reticulum lumen	74/839	311/19594	1.30E-34	2.84E-32	2.28E-32
CC	GO:0005604	basement membrane	35/839	95/19594	6.68E-24	9.68E-22	7.80E-22
CC	GO:0005581	collagen trimer	32/839	86/19594	4.04E-22	4.40E-20	3.54E-20
MF	GO:0005201	extracellular matrix structural constituent	73/803	172/18410	1.88E-53	1.36E-50	1.19E-50
MF	GO:0005178	integrin binding	52/803	156/18410	4.58E-32	1.66E-29	1.45E-29
MF	GO:0005518	collagen binding	35/803	68/18410	8.37E-30	2.02E-27	1.77E-27
MF	GO:0030020	extracellular matrix structural constituent conferring tensile strength	23/803	41/18410	3.64E-21	6.59E-19	5.78E-19
KEGG	hsa04510	Focal adhesion	46/345	201/8164	5.90E-22	1.65E-19	1.37E-19
KEGG	hsa04512	ECM-receptor interaction	29/345	88/8164	6.99E-19	9.78E-17	8.13E-17
KEGG	hsa04151	PI3K-Akt signaling pathway	50/345	354/8164	1.81E-14	1.69E-12	1.41E-12
KEGG	hsa04974	Protein digestion and absorption	26/345	103/8164	6.46E-14	4.52E-12	3.76E-12
