# Supplementary material for: A novel mitochondrial metabolism-related gene signature for predicting the prognosis of oesophageal squamous cell carcinoma
Source: Aging (Albany NY). 2024 Jun 5;16(11):9649–79. doi: 10.18632/aging.205892 (PMC11210263; doi:10.18632/aging.205892)
Supplement: Supplementary Table 6 [file aging-16-205892-s005.docx]

**Supplementary Table 6. DEGs of Cox Multifactorial Model.**

|  | **baseMean** | **log2FoldChange** | **lfcSE** | **stat** | **pvalue** | **padj** |
| --- | --- | --- | --- | --- | --- | --- |
| TDRD1 | 188.7155 | -4.80922 | 0.615017 | -7.81966 | 5.30E-15 | 9.57E-11 |
| CLDN19 | 38.94218 | -3.40701 | 0.499929 | -6.81499 | 9.43E-12 | 8.51E-08 |
| PIWIL2 | 237.3582 | -3.16333 | 0.501303 | -6.31023 | 2.79E-10 | 1.68E-06 |
| MUC19 | 68.73046 | -3.57018 | 0.573751 | -6.22253 | 4.89E-10 | 2.21E-06 |
| LHX8 | 43.31985 | -3.01833 | 0.493087 | -6.1213 | 9.28E-10 | 3.35E-06 |
| KIF1A | 719.4272 | -3.44773 | 0.570668 | -6.04157 | 1.53E-09 | 4.59E-06 |
| PADI2 | 1056.299 | -2.39455 | 0.406954 | -5.88408 | 4.00E-09 | 9.06E-06 |
| FOXJ1 | 210.4569 | -3.45055 | 0.586454 | -5.88374 | 4.01E-09 | 9.06E-06 |
| SYNE4 | 115.9978 | -1.96494 | 0.336589 | -5.8378 | 5.29E-09 | 1.06E-05 |
| HCAR1 | 53.89931 | -2.72912 | 0.471739 | -5.78523 | 7.24E-09 | 1.31E-05 |
| FABP4 | 1482.349 | 2.898314 | 0.506666 | 5.720363 | 1.06E-08 | 1.75E-05 |
| DLK1 | 14.07368 | -3.22469 | 0.574092 | -5.61701 | 1.94E-08 | 2.92E-05 |
| FOXN4 | 14.06309 | -3.49971 | 0.63778 | -5.48733 | 4.08E-08 | 5.67E-05 |
| TEX15 | 274.1329 | -3.09963 | 0.573946 | -5.40056 | 6.64E-08 | 8.00E-05 |
| CPLX2 | 88.22768 | -3.24974 | 0.601463 | -5.40307 | 6.55E-08 | 8.00E-05 |
| C5orf46 | 84.58969 | 2.295007 | 0.426686 | 5.378681 | 7.50E-08 | 8.47E-05 |
| ADH1C | 838.5294 | -3.84236 | 0.724715 | -5.30189 | 1.15E-07 | 0.000122 |
| MUC6 | 89.77174 | -2.44607 | 0.462495 | -5.28886 | 1.23E-07 | 0.000124 |
| ZNF541 | 48.93292 | -2.16966 | 0.411391 | -5.27395 | 1.34E-07 | 0.000127 |
| CHGA | 34.67742 | -2.16073 | 0.416784 | -5.1843 | 2.17E-07 | 0.00019 |
| NPFFR2 | 47.35114 | -3.31716 | 0.641934 | -5.16745 | 2.37E-07 | 0.000195 |
| SOST | 348.0779 | -2.96155 | 0.580579 | -5.10103 | 3.38E-07 | 0.000265 |
| ZNF486 | 382.8934 | -1.99855 | 0.392541 | -5.09131 | 3.56E-07 | 0.000268 |
| CDH12 | 140.8738 | -3.64069 | 0.72327 | -5.03365 | 4.81E-07 | 0.000315 |
| KRT83 | 15.85817 | -2.90064 | 0.576598 | -5.0306 | 4.89E-07 | 0.000315 |
| RNF182 | 136.7803 | -2.34466 | 0.464908 | -5.04328 | 4.58E-07 | 0.000315 |
| ZNF726 | 141.711 | -1.87043 | 0.371717 | -5.03186 | 4.86E-07 | 0.000315 |
| CH25H | 490.7974 | 1.951169 | 0.390325 | 4.998835 | 5.77E-07 | 0.000359 |
| ADAM22 | 378.1211 | -1.79178 | 0.363728 | -4.92616 | 8.39E-07 | 0.000505 |
| IRS4 | 59.13369 | -2.94914 | 0.601807 | -4.90048 | 9.56E-07 | 0.000557 |
| PTPRT | 230.3231 | 2.962489 | 0.612066 | 4.840146 | 1.30E-06 | 0.000732 |
| PLEKHH1 | 1195.655 | -1.00506 | 0.210319 | -4.77873 | 1.76E-06 | 0.000937 |
| PDIA2 | 30.56531 | -1.67982 | 0.353546 | -4.75133 | 2.02E-06 | 0.001026 |
| SLC10A5 | 34.32919 | -1.28463 | 0.270507 | -4.74896 | 2.04E-06 | 0.001026 |
| BIRC7 | 19.66077 | 2.347033 | 0.496908 | 4.723271 | 2.32E-06 | 0.001101 |
| MEX3A | 1869.679 | -1.78741 | 0.378329 | -4.72449 | 2.31E-06 | 0.001101 |
| CCL18 | 2140.632 | 2.123542 | 0.450049 | 4.718469 | 2.38E-06 | 0.001101 |
| ACTL8 | 222.5694 | -3.10732 | 0.66164 | -4.69639 | 2.65E-06 | 0.001154 |
| SHISAL1 | 789.4937 | 1.744233 | 0.371607 | 4.693758 | 2.68E-06 | 0.001154 |
| SAMD13 | 35.38482 | -1.36681 | 0.290613 | -4.70318 | 2.56E-06 | 0.001154 |
| SIK1 | 454.0263 | 1.748144 | 0.373959 | 4.674697 | 2.94E-06 | 0.001237 |
| HPN | 36.73583 | -2.06022 | 0.44155 | -4.66589 | 3.07E-06 | 0.001261 |
| FOXH1 | 20.02261 | -1.69872 | 0.36495 | -4.65467 | 3.25E-06 | 0.001297 |
| ZBBX | 8.526207 | -2.71639 | 0.584044 | -4.651 | 3.30E-06 | 0.001297 |
| CYRIA | 887.3769 | 1.104617 | 0.238195 | 4.637442 | 3.53E-06 | 0.001356 |
| WNK4 | 185.2502 | -1.80188 | 0.39333 | -4.58108 | 4.63E-06 | 0.001652 |
| MAEL | 32.8118 | -1.99651 | 0.435321 | -4.58629 | 4.51E-06 | 0.001652 |
| CDH4 | 266.0094 | 1.930843 | 0.422968 | 4.564986 | 5.00E-06 | 0.001702 |
| IL6 | 519.6376 | 1.873659 | 0.411514 | 4.553084 | 5.29E-06 | 0.001768 |
| ADCY8 | 13.59344 | -2.75097 | 0.606714 | -4.53421 | 5.78E-06 | 0.001899 |
| CACNA1D | 185.4119 | -1.54209 | 0.34267 | -4.50022 | 6.79E-06 | 0.002078 |
| SLITRK1 | 11.16793 | -2.59455 | 0.576527 | -4.5003 | 6.79E-06 | 0.002078 |
| NSUN7 | 223.5262 | -1.48053 | 0.328521 | -4.50666 | 6.59E-06 | 0.002078 |
| FTCD | 22.02014 | -1.5344 | 0.342386 | -4.48149 | 7.41E-06 | 0.002195 |
| TENT5D | 9.633093 | -3.33612 | 0.746436 | -4.4694 | 7.84E-06 | 0.002285 |
| PRDM9 | 13.47102 | -3.13776 | 0.703753 | -4.4586 | 8.25E-06 | 0.002328 |
| MYO18B | 155.019 | -2.22017 | 0.500793 | -4.43331 | 9.28E-06 | 0.002579 |
| SPATA21 | 18.71975 | -1.65357 | 0.373713 | -4.42471 | 9.66E-06 | 0.002643 |
| PDE10A | 724.4564 | -1.45641 | 0.330517 | -4.40647 | 1.05E-05 | 0.002752 |
| PABPC4L | 289.7331 | -1.61276 | 0.367084 | -4.39345 | 1.12E-05 | 0.002879 |
| ABCC2 | 264.4043 | -1.55013 | 0.353366 | -4.38675 | 1.15E-05 | 0.00291 |
| CCNB3 | 57.38309 | -1.48673 | 0.339514 | -4.37901 | 1.19E-05 | 0.00291 |
| SCN3B | 245.31 | 1.470166 | 0.335699 | 4.379416 | 1.19E-05 | 0.00291 |
| GOLGA6L7 | 46.10354 | -2.61029 | 0.596042 | -4.37938 | 1.19E-05 | 0.00291 |
| STX11 | 1051.546 | 1.101428 | 0.252693 | 4.358756 | 1.31E-05 | 0.003109 |
| TRIM54 | 34.51011 | -2.24783 | 0.51819 | -4.33785 | 1.44E-05 | 0.003366 |
| TEPP | 56.2105 | 1.710928 | 0.395307 | 4.328103 | 1.50E-05 | 0.003439 |
| GABRB2 | 96.33734 | -2.29761 | 0.531905 | -4.31958 | 1.56E-05 | 0.00353 |
| CTCFL | 28.7542 | -2.57294 | 0.59689 | -4.31057 | 1.63E-05 | 0.003609 |
| TMEM235 | 6.918932 | -2.44508 | 0.567405 | -4.30923 | 1.64E-05 | 0.003609 |
| MSH4 | 19.49352 | -1.73825 | 0.40437 | -4.29866 | 1.72E-05 | 0.003722 |
| ACTL6B | 11.00336 | -1.8815 | 0.440833 | -4.26804 | 1.97E-05 | 0.004013 |
| LCT | 21.30845 | -1.86296 | 0.436712 | -4.26587 | 1.99E-05 | 0.004013 |
| CHAD | 44.45663 | -1.36309 | 0.319107 | -4.27158 | 1.94E-05 | 0.004013 |
| THSD8 | 6.091032 | -1.96354 | 0.460394 | -4.26492 | 2.00E-05 | 0.004013 |
| ZNF385C | 191.4394 | -1.41021 | 0.330986 | -4.26063 | 2.04E-05 | 0.004046 |
| TENM1 | 472.3659 | -2.1847 | 0.513504 | -4.25451 | 2.10E-05 | 0.004113 |
| CT55 | 4.362748 | -2.71894 | 0.641221 | -4.24025 | 2.23E-05 | 0.004245 |
| IL17RB | 264.3216 | -1.03509 | 0.244292 | -4.23711 | 2.26E-05 | 0.004249 |
| CREG2 | 606.6367 | 1.752628 | 0.413809 | 4.235353 | 2.28E-05 | 0.004249 |
| MORN3 | 141.247 | -1.41675 | 0.335038 | -4.22862 | 2.35E-05 | 0.00429 |
| TMEM45A | 6779.402 | 1.55673 | 0.368347 | 4.22626 | 2.38E-05 | 0.004292 |
| LRRC3B | 21.10684 | -2.41786 | 0.572649 | -4.22223 | 2.42E-05 | 0.004326 |
| C1QL1 | 558.088 | 1.680054 | 0.399702 | 4.203263 | 2.63E-05 | 0.004614 |
| SPDYC | 65.76516 | -2.34767 | 0.55902 | -4.19962 | 2.67E-05 | 0.004622 |
| BRDT | 287.0581 | -2.77496 | 0.662196 | -4.19054 | 2.78E-05 | 0.004742 |
| SCN2A | 320.4552 | -2.1972 | 0.526801 | -4.17083 | 3.03E-05 | 0.005123 |
| RPS6KA6 | 199.6593 | -1.9992 | 0.480571 | -4.16005 | 3.18E-05 | 0.005225 |
| TBR1 | 8.102415 | -1.78415 | 0.428562 | -4.16312 | 3.14E-05 | 0.005225 |
| TRIM71 | 14.84877 | -2.04661 | 0.491948 | -4.16021 | 3.18E-05 | 0.005225 |
| SLC23A3 | 44.9146 | -1.13404 | 0.272855 | -4.15619 | 3.24E-05 | 0.005266 |
| ODAPH | 29.98528 | -1.77082 | 0.426793 | -4.14914 | 3.34E-05 | 0.005288 |
| LAMB4 | 79.23778 | -1.65402 | 0.39906 | -4.1448 | 3.40E-05 | 0.005342 |
| ZNF208 | 86.21947 | -1.83278 | 0.444779 | -4.12065 | 3.78E-05 | 0.005833 |
| DCX | 10.29484 | -2.03402 | 0.49556 | -4.10449 | 4.05E-05 | 0.006203 |
| STARD6 | 8.89454 | -1.50874 | 0.36889 | -4.08995 | 4.31E-05 | 0.006495 |
| TRIM50 | 6.507748 | -1.5671 | 0.383561 | -4.08567 | 4.39E-05 | 0.006561 |
| APOA5 | 2.363221 | -2.70303 | 0.664073 | -4.07039 | 4.69E-05 | 0.006721 |
| NR5A1 | 14.45503 | -1.95437 | 0.479567 | -4.07528 | 4.60E-05 | 0.006721 |
| DCDC2 | 22.16426 | -1.64734 | 0.405052 | -4.06698 | 4.76E-05 | 0.006721 |
| FAM186A | 42.10036 | -1.31778 | 0.323909 | -4.06837 | 4.73E-05 | 0.006721 |
| TNFSF11 | 86.88359 | 1.453667 | 0.357919 | 4.061445 | 4.88E-05 | 0.006829 |
| DDX53 | 16.45937 | -3.31416 | 0.817478 | -4.05413 | 5.03E-05 | 0.006985 |
| DCC | 73.4709 | -1.91451 | 0.472625 | -4.0508 | 5.10E-05 | 0.006985 |
| ZNF98 | 19.41874 | -2.34868 | 0.579704 | -4.05152 | 5.09E-05 | 0.006985 |
| TRIM72 | 47.34539 | -1.65545 | 0.410801 | -4.02981 | 5.58E-05 | 0.007525 |
| CXXC4 | 169.7651 | -1.60558 | 0.398787 | -4.02615 | 5.67E-05 | 0.007586 |
| LYNX1 | 1122.781 | 1.207753 | 0.300214 | 4.022973 | 5.75E-05 | 0.007633 |
| CFAP65 | 9.704292 | -1.73683 | 0.432769 | -4.0133 | 5.99E-05 | 0.007895 |
| SCGB1A1 | 27.18889 | -2.49175 | 0.621238 | -4.01094 | 6.05E-05 | 0.007916 |
| TAT | 11.42806 | -1.84917 | 0.463862 | -3.98648 | 6.71E-05 | 0.008354 |
| ZNF626 | 343.8618 | -1.70784 | 0.430135 | -3.97048 | 7.17E-05 | 0.008874 |
| KCNV1 | 13.52706 | -2.6311 | 0.664437 | -3.9599 | 7.50E-05 | 0.009161 |
| RAB39A | 73.32615 | -1.54363 | 0.390314 | -3.95485 | 7.66E-05 | 0.009161 |
| PPM1E | 80.62905 | -1.4469 | 0.3661 | -3.95221 | 7.74E-05 | 0.009202 |
| CPNE6 | 10.65287 | -1.63518 | 0.415508 | -3.93537 | 8.31E-05 | 0.009403 |
| COL20A1 | 6.165427 | -1.72912 | 0.438538 | -3.94292 | 8.05E-05 | 0.009403 |
| SECTM1 | 2322.552 | 1.113228 | 0.283002 | 3.933644 | 8.37E-05 | 0.009403 |
| UGT3A1 | 14.04277 | -2.83036 | 0.718363 | -3.94002 | 8.15E-05 | 0.009403 |
| STMND1 | 10.61954 | -2.07252 | 0.526715 | -3.9348 | 8.33E-05 | 0.009403 |
| CPAMD8 | 511.6626 | -1.41555 | 0.360447 | -3.92719 | 8.59E-05 | 0.009408 |
| AR | 216.1351 | -1.28782 | 0.327701 | -3.92987 | 8.50E-05 | 0.009408 |
| ZNF90 | 154.5563 | -1.69315 | 0.431399 | -3.9248 | 8.68E-05 | 0.009445 |
| NTSR1 | 86.79974 | -1.95087 | 0.497306 | -3.92288 | 8.75E-05 | 0.009461 |
| CORO2B | 177.9686 | 1.293044 | 0.329731 | 3.921516 | 8.80E-05 | 0.009461 |
| CHDH | 384.3169 | -1.33148 | 0.3402 | -3.91381 | 9.08E-05 | 0.009541 |
| ZNF716 | 26.4924 | -2.94915 | 0.753489 | -3.91399 | 9.08E-05 | 0.009541 |
| ZBTB8B | 35.38819 | -1.73945 | 0.444143 | -3.91641 | 8.99E-05 | 0.009541 |
| CAMK2B | 181.784 | -1.60016 | 0.409486 | -3.90773 | 9.32E-05 | 0.009681 |
| WDR87 | 20.56623 | -1.79875 | 0.460388 | -3.90704 | 9.34E-05 | 0.009681 |
| SLC12A1 | 13.76456 | -2.01473 | 0.517437 | -3.89368 | 9.87E-05 | 0.009859 |
| CYP4B1 | 389.7229 | -2.18642 | 0.561575 | -3.89338 | 9.89E-05 | 0.009859 |
| HHIPL2 | 57.36934 | -1.62233 | 0.416965 | -3.89081 | 9.99E-05 | 0.009859 |
| MCHR2 | 11.10376 | -2.25079 | 0.578998 | -3.88738 | 0.000101 | 0.009859 |
| RALYL | 6.760573 | -2.49436 | 0.641732 | -3.88692 | 0.000102 | 0.009859 |
| TBC1D26 | 2.450282 | -2.01107 | 0.517167 | -3.88863 | 0.000101 | 0.009859 |
| TEX35 | 9.605755 | -1.95166 | 0.503103 | -3.87925 | 0.000105 | 0.010121 |
| WDR64 | 17.56857 | -1.84644 | 0.476446 | -3.87544 | 0.000106 | 0.010172 |
| F2 | 8.695325 | -1.79642 | 0.463506 | -3.87573 | 0.000106 | 0.010172 |
| KIF25 | 28.43684 | -1.71303 | 0.442778 | -3.86883 | 0.000109 | 0.010377 |
| SKIDA1 | 105.9559 | -1.03641 | 0.268103 | -3.86573 | 0.000111 | 0.010377 |
| NMNAT2 | 243.7444 | 1.073433 | 0.278175 | 3.858846 | 0.000114 | 0.010499 |
| CABYR | 885.1805 | -1.63378 | 0.424932 | -3.8448 | 0.000121 | 0.010883 |
| KLK2 | 17.75898 | -1.98099 | 0.515287 | -3.84444 | 0.000121 | 0.010883 |
| GJA9 | 3.016371 | -1.38956 | 0.3622 | -3.83644 | 0.000125 | 0.011107 |
| ZNF738 | 534.3637 | -1.27424 | 0.332379 | -3.8337 | 0.000126 | 0.011123 |
| PPP1R3C | 1461.532 | -1.85323 | 0.483718 | -3.83123 | 0.000128 | 0.01118 |
| NUTM1 | 20.57136 | -1.40557 | 0.367088 | -3.82896 | 0.000129 | 0.011229 |
| CD209 | 501.0085 | 1.488734 | 0.389621 | 3.820985 | 0.000133 | 0.011488 |
| CEL | 1924.26 | -2.04731 | 0.536401 | -3.81676 | 0.000135 | 0.011615 |
| PALM3 | 59.30342 | -1.48322 | 0.388812 | -3.81474 | 0.000136 | 0.011615 |
| ESRRB | 34.54914 | -1.35908 | 0.356408 | -3.81327 | 0.000137 | 0.01163 |
| VWA2 | 1128.69 | -1.42394 | 0.373697 | -3.8104 | 0.000139 | 0.011711 |
| ACSM3 | 124.2497 | -1.15988 | 0.304623 | -3.8076 | 0.00014 | 0.011789 |
| NPY5R | 2.583925 | -2.40484 | 0.633025 | -3.79896 | 0.000145 | 0.011985 |
| HSD17B13 | 34.70492 | -1.50851 | 0.397033 | -3.79945 | 0.000145 | 0.011985 |
| STRA6 | 2368.37 | 1.647127 | 0.434229 | 3.79322 | 0.000149 | 0.012 |
| TSPAN19 | 5.814859 | -1.62817 | 0.429199 | -3.79351 | 0.000149 | 0.012 |
| SULT1E1 | 187.6688 | -2.07658 | 0.54837 | -3.78683 | 0.000153 | 0.012035 |
| STXBP5L | 76.20858 | -1.79696 | 0.475283 | -3.78081 | 0.000156 | 0.012223 |
| NWD1 | 141.2461 | -1.53917 | 0.40773 | -3.77497 | 0.00016 | 0.012406 |
| NPPC | 121.8458 | -1.99587 | 0.528983 | -3.77304 | 0.000161 | 0.012449 |
| SORCS2 | 820.1622 | 1.114263 | 0.295445 | 3.771474 | 0.000162 | 0.012474 |
| ADRA1B | 46.4907 | 1.608085 | 0.426871 | 3.767148 | 0.000165 | 0.012638 |
| TCHH | 1092.777 | 1.787595 | 0.475469 | 3.759648 | 0.00017 | 0.012968 |
| UPK1A | 68.55919 | -1.80337 | 0.47991 | -3.75773 | 0.000171 | 0.013013 |
| TMEM170B | 384.491 | -1.13978 | 0.303896 | -3.75055 | 0.000176 | 0.013327 |
| DAND5 | 20.84916 | -1.24721 | 0.333268 | -3.74238 | 0.000182 | 0.01354 |
| EPHA10 | 97.29172 | -1.45549 | 0.389073 | -3.74093 | 0.000183 | 0.01354 |
| MEIG1 | 21.40967 | -1.16913 | 0.31294 | -3.73594 | 0.000187 | 0.013566 |
| HILPDA | 1648.868 | -1.13056 | 0.303349 | -3.72694 | 0.000194 | 0.014004 |
| L1TD1 | 51.36382 | -1.84036 | 0.494237 | -3.72364 | 0.000196 | 0.014132 |
| C5AR2 | 201.8819 | 1.306981 | 0.351629 | 3.716927 | 0.000202 | 0.014174 |
| ZNF66 | 157.468 | -1.43831 | 0.386747 | -3.71898 | 0.0002 | 0.014174 |
| CA8 | 98.68211 | -1.52316 | 0.40957 | -3.71892 | 0.0002 | 0.014174 |
| SP9 | 61.53823 | -1.66781 | 0.448635 | -3.71751 | 0.000201 | 0.014174 |
| MYBPC2 | 21.49159 | -1.42322 | 0.38316 | -3.71443 | 0.000204 | 0.014224 |
| SPEM2 | 2.765096 | -1.88927 | 0.508678 | -3.71407 | 0.000204 | 0.014224 |
| SLC9C2 | 4.512758 | -2.21079 | 0.596885 | -3.70388 | 0.000212 | 0.014527 |
| OBP2B | 14.59309 | -2.51683 | 0.679237 | -3.70537 | 0.000211 | 0.014527 |
| MARCOL | 6.808136 | -1.95059 | 0.526597 | -3.70415 | 0.000212 | 0.014527 |
| JAKMIP2 | 263.055 | 1.735881 | 0.468903 | 3.702003 | 0.000214 | 0.01458 |
| LAYN | 1086.736 | 1.192603 | 0.322254 | 3.700821 | 0.000215 | 0.014593 |
| ABCA4 | 665.9714 | -2.02423 | 0.547215 | -3.69914 | 0.000216 | 0.014635 |
| ZNF737 | 442.0955 | -1.41301 | 0.382323 | -3.69587 | 0.000219 | 0.01471 |
| ZNF492 | 33.73024 | -1.81341 | 0.491087 | -3.69263 | 0.000222 | 0.014739 |
| UNC13A | 108.4888 | -1.45405 | 0.393902 | -3.69141 | 0.000223 | 0.014756 |
| SIM1 | 15.21574 | -2.51868 | 0.682836 | -3.68857 | 0.000226 | 0.014867 |
| ESPNL | 113.7995 | -1.54146 | 0.418499 | -3.6833 | 0.00023 | 0.015078 |
| MYBPC1 | 32.80726 | -2.29821 | 0.625393 | -3.67483 | 0.000238 | 0.01536 |
| TCF24 | 56.68927 | -1.11796 | 0.304179 | -3.67536 | 0.000238 | 0.01536 |
| ONECUT2 | 422.6454 | -1.24889 | 0.340309 | -3.66986 | 0.000243 | 0.01547 |
| ATP6V0D2 | 106.7566 | 1.400887 | 0.381758 | 3.669571 | 0.000243 | 0.01547 |
| MYBPH | 7.14524 | -1.73547 | 0.473667 | -3.66389 | 0.000248 | 0.015717 |
| NTN3 | 50.95257 | -1.32866 | 0.362681 | -3.66344 | 0.000249 | 0.015717 |
| GTSF1 | 908.2286 | -2.15411 | 0.588202 | -3.6622 | 0.00025 | 0.015721 |
| SEMA5B | 416.7818 | 1.345965 | 0.368027 | 3.657241 | 0.000255 | 0.015825 |
| PLAAT4 | 1451.401 | 1.186358 | 0.32432 | 3.65798 | 0.000254 | 0.015825 |
| FOXR1 | 4.108427 | -2.06788 | 0.565702 | -3.65542 | 0.000257 | 0.015849 |
| GABBR2 | 36.96099 | -1.50903 | 0.413565 | -3.64884 | 0.000263 | 0.016063 |
| TUBB2A | 2928.042 | 1.089648 | 0.299253 | 3.641225 | 0.000271 | 0.016338 |
| GRB14 | 115.919 | -1.30814 | 0.359723 | -3.63653 | 0.000276 | 0.016528 |
| SYCP2L | 51.45499 | -1.66914 | 0.459127 | -3.63547 | 0.000277 | 0.016542 |
| HOATZ | 26.30122 | 1.550664 | 0.426992 | 3.631597 | 0.000282 | 0.016681 |
| EVX1 | 3.944596 | -1.99956 | 0.551556 | -3.62531 | 0.000289 | 0.017037 |
| CHRM5 | 26.85268 | -1.18585 | 0.327736 | -3.6183 | 0.000297 | 0.017181 |
| RGS9BP | 35.40183 | -1.21783 | 0.336435 | -3.6198 | 0.000295 | 0.017181 |
| PSORS1C2 | 141.8066 | 1.689442 | 0.466941 | 3.618105 | 0.000297 | 0.017181 |
| ORM1 | 9.414287 | -2.38125 | 0.657993 | -3.61896 | 0.000296 | 0.017181 |
| RNF17 | 14.5057 | -1.41343 | 0.391256 | -3.61254 | 0.000303 | 0.017357 |
| ADRA2A | 310.8005 | 1.449995 | 0.401469 | 3.611721 | 0.000304 | 0.017357 |
| PCSK4 | 291.7043 | -1.12387 | 0.311452 | -3.60849 | 0.000308 | 0.01744 |
| CRACD | 335.8493 | -1.16584 | 0.323866 | -3.59975 | 0.000319 | 0.01798 |
| CLUL1 | 24.49127 | -1.51997 | 0.42343 | -3.58966 | 0.000331 | 0.018452 |
| LBX1 | 17.26847 | 2.614003 | 0.72846 | 3.588394 | 0.000333 | 0.018452 |
| PTPRD | 1066.648 | -1.57633 | 0.439314 | -3.58817 | 0.000333 | 0.018452 |
| CCER2 | 19.25333 | -1.2854 | 0.358083 | -3.58967 | 0.000331 | 0.018452 |
| SYDE2 | 441.7691 | -1.10669 | 0.308777 | -3.5841 | 0.000338 | 0.018514 |
| CLDN5 | 963.2661 | 1.152695 | 0.321891 | 3.581008 | 0.000342 | 0.018566 |
| EFNA2 | 34.32825 | -1.31953 | 0.368978 | -3.57619 | 0.000349 | 0.018855 |
| SVOP | 12.09424 | -1.62462 | 0.455018 | -3.57044 | 0.000356 | 0.019141 |
| CEACAM19 | 2934.507 | 1.158854 | 0.325152 | 3.564033 | 0.000365 | 0.019459 |
| DRD5 | 27.58352 | 1.437045 | 0.403813 | 3.558686 | 0.000373 | 0.019749 |
| PRSS41 | 11.71656 | -1.87399 | 0.529762 | -3.53741 | 0.000404 | 0.020973 |
| SLC4A4 | 102.6816 | -1.17607 | 0.33277 | -3.53419 | 0.000409 | 0.021109 |
| OR51B5 | 13.91251 | -2.15332 | 0.609715 | -3.53168 | 0.000413 | 0.02125 |
| LORICRIN | 118.4723 | 1.92461 | 0.545702 | 3.526852 | 0.000421 | 0.021519 |
| GRK1 | 7.12309 | 1.902952 | 0.53982 | 3.525157 | 0.000423 | 0.021596 |
| TSGA10IP | 11.59605 | -1.80568 | 0.512719 | -3.52177 | 0.000429 | 0.021751 |
| ACBD7 | 225.3174 | -1.2012 | 0.34125 | -3.51999 | 0.000432 | 0.021774 |
| SYNPO2L | 109.1558 | -1.52663 | 0.436319 | -3.49889 | 0.000467 | 0.023121 |
| CCDC166 | 6.473789 | -1.61715 | 0.463025 | -3.49258 | 0.000478 | 0.023379 |
| GPD1 | 47.24645 | -1.03381 | 0.296321 | -3.4888 | 0.000485 | 0.023559 |
| PDE6A | 59.16227 | -1.22011 | 0.349895 | -3.48709 | 0.000488 | 0.023647 |
| ITLN1 | 11.75576 | -1.74759 | 0.501776 | -3.4828 | 0.000496 | 0.023901 |
| FXYD4 | 10.53621 | -1.89429 | 0.544345 | -3.47995 | 0.000502 | 0.024028 |
| DNAH12 | 27.95428 | -1.25896 | 0.362088 | -3.47694 | 0.000507 | 0.024045 |
| KIAA1210 | 15.61582 | -1.55872 | 0.448214 | -3.47763 | 0.000506 | 0.024045 |
| ARGFX | 9.799282 | -1.94163 | 0.558963 | -3.47363 | 0.000513 | 0.024144 |
| LCE1C | 57.25679 | 1.507789 | 0.433978 | 3.474343 | 0.000512 | 0.024144 |
| FRMPD2 | 6.706736 | -1.52339 | 0.439344 | -3.46741 | 0.000526 | 0.024556 |
| CNGB1 | 1914.757 | 1.795099 | 0.518054 | 3.465083 | 0.00053 | 0.024677 |
| EPHA8 | 31.54252 | -1.72274 | 0.498167 | -3.45815 | 0.000544 | 0.024935 |
| AP3B2 | 487.513 | -1.35829 | 0.39272 | -3.45867 | 0.000543 | 0.024935 |
| RFX4 | 12.90566 | -2.11992 | 0.612925 | -3.4587 | 0.000543 | 0.024935 |
| LINC00514 | 54.06655 | -1.57081 | 0.453966 | -3.4602 | 0.00054 | 0.024935 |
| TREH | 22.13862 | -1.2288 | 0.355428 | -3.45724 | 0.000546 | 0.024956 |
| SNCB | 108.0567 | 1.617664 | 0.468084 | 3.45593 | 0.000548 | 0.025014 |
| NR2E1 | 129.2131 | -1.75158 | 0.507058 | -3.45439 | 0.000552 | 0.025031 |
| COL21A1 | 400.2878 | -1.51787 | 0.440262 | -3.44765 | 0.000565 | 0.025331 |
| CCDC27 | 14.50562 | -1.27293 | 0.36947 | -3.4453 | 0.00057 | 0.025441 |
| ADAM20 | 24.77413 | -1.00638 | 0.292231 | -3.44379 | 0.000574 | 0.025515 |
| VASH2 | 572.4123 | -1.36875 | 0.397655 | -3.44206 | 0.000577 | 0.025515 |
| CYP2A7 | 7.482179 | -1.35526 | 0.393752 | -3.4419 | 0.000578 | 0.025515 |
| TEKT5 | 9.642158 | -1.17764 | 0.342667 | -3.4367 | 0.000589 | 0.025754 |
| WNT6 | 134.7933 | -1.3927 | 0.405322 | -3.43603 | 0.00059 | 0.025755 |
| TFAP2B | 82.21113 | -2.30999 | 0.673701 | -3.4288 | 0.000606 | 0.025885 |
| MDGA2 | 41.83931 | -2.38174 | 0.694841 | -3.42774 | 0.000609 | 0.025885 |
| EVX2 | 7.809076 | -1.83803 | 0.536386 | -3.4267 | 0.000611 | 0.025885 |
| CRACR2B | 345.9533 | -1.09798 | 0.320207 | -3.42896 | 0.000606 | 0.025885 |
| ANKRD45 | 18.28247 | -1.37004 | 0.399864 | -3.42628 | 0.000612 | 0.025885 |
| DMRT1 | 29.94884 | -1.67466 | 0.489101 | -3.42395 | 0.000617 | 0.025887 |
| PLG | 5.65585 | -1.67708 | 0.491105 | -3.4149 | 0.000638 | 0.026253 |
| FBN3 | 198.0343 | -1.86597 | 0.546227 | -3.41611 | 0.000635 | 0.026253 |
| SLC7A2 | 1631.77 | -1.30605 | 0.382705 | -3.41268 | 0.000643 | 0.026348 |
| UBE2U | 16.1374 | -2.16717 | 0.634931 | -3.41324 | 0.000642 | 0.026348 |
| LHFPL3 | 10.5939 | -1.98657 | 0.582439 | -3.41078 | 0.000648 | 0.02647 |
| ZNF676 | 29.20378 | -1.8364 | 0.539476 | -3.40404 | 0.000664 | 0.02677 |
| MPPED1 | 113.246 | 2.1129 | 0.621364 | 3.400424 | 0.000673 | 0.026999 |
| ARHGDIG | 14.30002 | -1.5867 | 0.466689 | -3.3999 | 0.000674 | 0.026999 |
| DPYSL5 | 66.28267 | -1.74947 | 0.514761 | -3.3986 | 0.000677 | 0.027067 |
| PIWIL3 | 6.260067 | -1.81717 | 0.535192 | -3.39536 | 0.000685 | 0.027269 |
| PAH | 40.3442 | -1.65578 | 0.488475 | -3.3897 | 0.0007 | 0.027595 |
| FETUB | 614.3114 | 2.182392 | 0.644284 | 3.387313 | 0.000706 | 0.027715 |
| COL9A1 | 31.64852 | -1.45528 | 0.430018 | -3.38423 | 0.000714 | 0.027738 |
| SEPTIN14 | 10.08821 | -2.12372 | 0.627807 | -3.38276 | 0.000718 | 0.027738 |
| DYDC2 | 22.4825 | -1.50042 | 0.443885 | -3.3802 | 0.000724 | 0.027897 |
| FCRLA | 75.73903 | 1.433147 | 0.424061 | 3.379579 | 0.000726 | 0.0279 |
| C12orf50 | 4.287244 | -1.45428 | 0.431243 | -3.3723 | 0.000745 | 0.028527 |
| DNAI3 | 31.17636 | -1.06662 | 0.31653 | -3.36972 | 0.000752 | 0.028674 |
| KLHL4 | 209.3374 | 1.596783 | 0.474013 | 3.368648 | 0.000755 | 0.028725 |
| GJC3 | 13.38469 | -1.33659 | 0.397776 | -3.36015 | 0.000779 | 0.029376 |
| COL19A1 | 42.15708 | -1.54523 | 0.460179 | -3.3579 | 0.000785 | 0.029555 |
| GRIA4 | 13.52093 | -1.2555 | 0.374216 | -3.35501 | 0.000794 | 0.02968 |
| EFCAB1 | 141.6771 | -1.12675 | 0.335944 | -3.35399 | 0.000797 | 0.029728 |
| AC011511.1 | 1.399302 | -1.57085 | 0.468987 | -3.34946 | 0.00081 | 0.029848 |
| GLB1L3 | 163.0606 | -1.76547 | 0.528145 | -3.34277 | 0.000829 | 0.030407 |
| C1QA | 4899.452 | 1.009131 | 0.301906 | 3.342538 | 0.00083 | 0.030407 |
| C1orf146 | 5.982998 | -1.19159 | 0.357 | -3.3378 | 0.000844 | 0.030644 |
| PVALB | 14.38482 | 1.45598 | 0.436386 | 3.336447 | 0.000849 | 0.030655 |
| AC139491.7 | 22.8493 | -2.02057 | 0.605533 | -3.33685 | 0.000847 | 0.030655 |
| PRKAG3 | 11.23505 | -1.579 | 0.473802 | -3.33261 | 0.00086 | 0.03075 |
| DIRAS2 | 176.8677 | -2.05318 | 0.616288 | -3.33153 | 0.000864 | 0.03075 |
| MAB21L2 | 6.573161 | -1.15961 | 0.348014 | -3.33208 | 0.000862 | 0.03075 |
| AIF1L | 2342.176 | -1.19335 | 0.358786 | -3.32608 | 0.000881 | 0.031133 |
| APOH | 7.205098 | -1.83027 | 0.551125 | -3.32098 | 0.000897 | 0.031585 |
| ZSCAN10 | 7.338875 | -1.52905 | 0.460361 | -3.32141 | 0.000896 | 0.031585 |
| HMX2 | 21.19088 | -1.83311 | 0.552814 | -3.31597 | 0.000913 | 0.03197 |
| SLC25A31 | 3.84533 | -1.75636 | 0.530791 | -3.30895 | 0.000936 | 0.032266 |
| PCDHA10 | 26.27437 | -1.41278 | 0.427009 | -3.30855 | 0.000938 | 0.032266 |
| CCDC177 | 155.7685 | 1.625111 | 0.490966 | 3.310025 | 0.000933 | 0.032266 |
| SNCG | 590.334 | -1.21686 | 0.367963 | -3.30702 | 0.000943 | 0.03232 |
| ZNF99 | 20.0846 | -1.80277 | 0.545926 | -3.30223 | 0.000959 | 0.032753 |
| SH3BGRL2 | 2202.438 | -1.2579 | 0.381149 | -3.30028 | 0.000966 | 0.032856 |
| ADGB | 7.780833 | -1.83085 | 0.554918 | -3.29932 | 0.000969 | 0.032907 |
| TRPV5 | 4.809941 | -1.86728 | 0.566239 | -3.29768 | 0.000975 | 0.032929 |
| PNPLA1 | 145.9951 | 1.401513 | 0.425016 | 3.297553 | 0.000975 | 0.032929 |
| LSMEM2 | 6.603475 | -1.08063 | 0.327954 | -3.29506 | 0.000984 | 0.033037 |
| ZNF682 | 187.4268 | -1.15873 | 0.351548 | -3.29607 | 0.00098 | 0.033037 |
| DNAH5 | 384.0159 | 1.21222 | 0.368119 | 3.29301 | 0.000991 | 0.033215 |
| C6orf118 | 11.00351 | -2.1778 | 0.661545 | -3.29199 | 0.000995 | 0.033215 |
| F13B | 2.203443 | -2.028 | 0.61702 | -3.28676 | 0.001013 | 0.033536 |
| ZFP42 | 33.27376 | -1.9679 | 0.59861 | -3.28745 | 0.001011 | 0.033536 |
| SATL1 | 9.460308 | -1.57811 | 0.480429 | -3.28481 | 0.001021 | 0.0337 |
| TNFSF18 | 239.689 | 1.565275 | 0.476766 | 3.283109 | 0.001027 | 0.033834 |
| ISM2 | 64.93453 | -1.31664 | 0.401708 | -3.2776 | 0.001047 | 0.034321 |
| PPP2R2B | 256.2193 | 1.345389 | 0.410699 | 3.275849 | 0.001053 | 0.034408 |
| HYDIN | 44.01007 | -1.37279 | 0.41907 | -3.2758 | 0.001054 | 0.034408 |
| BRINP2 | 13.55403 | -2.04503 | 0.625083 | -3.27161 | 0.001069 | 0.03476 |
| P2RX3 | 5.772067 | -1.46132 | 0.447568 | -3.26502 | 0.001095 | 0.035243 |
| DNAJC5G | 6.542919 | -1.86074 | 0.570505 | -3.26156 | 0.001108 | 0.035361 |
| SLC28A1 | 15.67485 | -1.51658 | 0.465216 | -3.25996 | 0.001114 | 0.035414 |
| GOLGA8S | 3.839899 | -1.51371 | 0.46429 | -3.26027 | 0.001113 | 0.035414 |
| TUBA3E | 12.63464 | -1.31188 | 0.402841 | -3.25658 | 0.001128 | 0.035671 |
| SFRP1 | 4467.41 | -1.81214 | 0.556832 | -3.25437 | 0.001136 | 0.035887 |
| FGG | 9.163479 | -2.08383 | 0.640798 | -3.25193 | 0.001146 | 0.035946 |
| INSM1 | 19.90256 | -1.42323 | 0.438014 | -3.24927 | 0.001157 | 0.035971 |
| CCKBR | 13.69167 | -1.96465 | 0.604738 | -3.24876 | 0.001159 | 0.035974 |
| PPP1R1B | 421.29 | -1.86438 | 0.574585 | -3.24475 | 0.001176 | 0.036092 |
| NKX2-1 | 23.18147 | -1.93263 | 0.595291 | -3.24652 | 0.001168 | 0.036092 |
| IL19 | 63.33866 | 1.290202 | 0.397796 | 3.243374 | 0.001181 | 0.036092 |
| SFTPA2 | 13.49547 | -1.82752 | 0.563957 | -3.24053 | 0.001193 | 0.036159 |
| GRM3 | 10.80142 | -1.13938 | 0.351882 | -3.23794 | 0.001204 | 0.036366 |
| ANGPT4 | 22.33198 | 1.065818 | 0.329587 | 3.233801 | 0.001222 | 0.03677 |
| CDX1 | 58.81173 | 1.245178 | 0.385214 | 3.232431 | 0.001227 | 0.03677 |
| RBM20 | 138.2702 | -1.15188 | 0.356407 | -3.23194 | 0.00123 | 0.03677 |
| PRSS37 | 3.722724 | -1.68776 | 0.52255 | -3.22986 | 0.001239 | 0.036978 |
| TRPC3 | 61.10221 | -1.06785 | 0.331361 | -3.22263 | 0.00127 | 0.037417 |
| PON1 | 14.03984 | -1.60033 | 0.496864 | -3.22086 | 0.001278 | 0.037503 |
| LGI1 | 21.33625 | -1.62478 | 0.504917 | -3.21791 | 0.001291 | 0.037503 |
| CRYAB | 4012.813 | 1.094401 | 0.3401 | 3.21788 | 0.001291 | 0.037503 |
| ETNPPL | 6.316322 | -1.92573 | 0.597982 | -3.22039 | 0.00128 | 0.037503 |
| CD3D | 324.6658 | 1.097553 | 0.341022 | 3.218422 | 0.001289 | 0.037503 |
| CELF3 | 13.04597 | -1.05891 | 0.32953 | -3.2134 | 0.001312 | 0.03785 |
| CYP3A43 | 6.945896 | -1.1756 | 0.366055 | -3.21155 | 0.00132 | 0.037958 |
| PCDHGB2 | 409.8035 | 1.183303 | 0.368492 | 3.211206 | 0.001322 | 0.037958 |
| COL2A1 | 136.8139 | -1.34085 | 0.418257 | -3.20579 | 0.001347 | 0.038314 |
| PERCC1 | 4.850506 | -1.72686 | 0.540211 | -3.19663 | 0.00139 | 0.038998 |
| SLIT1 | 65.15169 | 1.067171 | 0.333934 | 3.195751 | 0.001395 | 0.039057 |
| KLK1 | 265.2418 | 1.151322 | 0.360567 | 3.193086 | 0.001408 | 0.039298 |
| PIGR | 1580.109 | 1.902275 | 0.596006 | 3.191703 | 0.001414 | 0.039424 |
| KRTAP3-1 | 6.17144 | -2.11046 | 0.66155 | -3.19017 | 0.001422 | 0.039483 |
| WEE2 | 9.286426 | -1.09176 | 0.342644 | -3.18629 | 0.001441 | 0.039802 |
| MUC22 | 203.3053 | -2.10276 | 0.660802 | -3.18213 | 0.001462 | 0.040194 |
| ZNF695 | 126.4384 | -1.06023 | 0.333302 | -3.18098 | 0.001468 | 0.040271 |
| PAK3 | 105.0037 | -1.42337 | 0.44778 | -3.17872 | 0.001479 | 0.040485 |
| ALPI | 3.648116 | -2.23197 | 0.702544 | -3.17698 | 0.001488 | 0.040667 |
| CPS1 | 430.7106 | -1.32677 | 0.418626 | -3.16934 | 0.001528 | 0.041045 |
| CENPS-CORT | 4.33204 | -1.07289 | 0.338496 | -3.16958 | 0.001527 | 0.041045 |
| AARD | 34.21445 | -1.32944 | 0.420622 | -3.16066 | 0.001574 | 0.041692 |
| CLDN6 | 22.71069 | -1.51004 | 0.479237 | -3.15092 | 0.001628 | 0.042793 |
| NUP210L | 46.52052 | -1.32348 | 0.420369 | -3.14838 | 0.001642 | 0.042854 |
| HS3ST5 | 62.83867 | 1.925143 | 0.612056 | 3.145368 | 0.001659 | 0.042926 |
| SNTG1 | 5.364119 | -1.84901 | 0.58875 | -3.14057 | 0.001686 | 0.043059 |
| TAS1R1 | 17.33678 | -1.02131 | 0.325269 | -3.13989 | 0.00169 | 0.043059 |
| CARD11 | 1634.29 | 1.247162 | 0.396929 | 3.142027 | 0.001678 | 0.043059 |
| LCN8 | 3.211689 | -1.85572 | 0.590691 | -3.1416 | 0.00168 | 0.043059 |
| RGL3 | 97.5041 | -1.12054 | 0.356473 | -3.14341 | 0.00167 | 0.043059 |
| RLBP1 | 11.58184 | -1.42352 | 0.454497 | -3.13208 | 0.001736 | 0.04403 |
| NECAB2 | 132.3913 | -1.56494 | 0.500078 | -3.1294 | 0.001752 | 0.044135 |
| RDH12 | 530.8064 | 1.560166 | 0.498619 | 3.128975 | 0.001754 | 0.044135 |
| DRC7 | 7.106339 | -1.4409 | 0.460596 | -3.12835 | 0.001758 | 0.044154 |
| ZIM3 | 3.694331 | -1.89769 | 0.606923 | -3.12673 | 0.001768 | 0.044222 |
| NRXN1 | 197.2706 | -1.61887 | 0.518276 | -3.12356 | 0.001787 | 0.044578 |
| KRT39 | 7.624103 | -1.74312 | 0.558286 | -3.12227 | 0.001795 | 0.044712 |
| FAM237B | 11.13018 | -1.19572 | 0.383264 | -3.11984 | 0.001809 | 0.044958 |
| USH2A | 37.44015 | -1.29329 | 0.414698 | -3.11863 | 0.001817 | 0.045082 |
| CHAT | 26.76441 | 1.767659 | 0.567142 | 3.116786 | 0.001828 | 0.045302 |
| C6orf52 | 68.28135 | -1.07252 | 0.344641 | -3.112 | 0.001858 | 0.045808 |
| AC108941.3 | 3.196191 | -1.93626 | 0.622754 | -3.10919 | 0.001876 | 0.045978 |
| DACH1 | 216.8712 | -1.16453 | 0.375017 | -3.10528 | 0.001901 | 0.04634 |
| TMEM178B | 914.069 | -1.60336 | 0.516477 | -3.10442 | 0.001907 | 0.04635 |
| BEST2 | 44.46227 | -1.17561 | 0.378959 | -3.10222 | 0.001921 | 0.046571 |
| HCN4 | 6.206401 | -1.27321 | 0.411169 | -3.09657 | 0.001958 | 0.047021 |
| ACOT6 | 3.667697 | -1.47847 | 0.477574 | -3.0958 | 0.001963 | 0.047021 |
| VIPR2 | 28.55263 | -1.25982 | 0.407165 | -3.09414 | 0.001974 | 0.047224 |
| LBHD2 | 29.73965 | 1.60591 | 0.519393 | 3.091898 | 0.001989 | 0.047393 |
| HMX3 | 11.32508 | -1.90295 | 0.616035 | -3.08903 | 0.002008 | 0.047664 |
| LRRD1 | 13.677 | -1.28916 | 0.417529 | -3.08759 | 0.002018 | 0.047723 |
| ANKLE1 | 156.4458 | -1.11954 | 0.362942 | -3.08461 | 0.002038 | 0.047999 |
| SYT13 | 176.5661 | 1.532351 | 0.49711 | 3.082517 | 0.002053 | 0.048276 |
| ANKRD30B | 9.23186 | -2.08539 | 0.676951 | -3.08056 | 0.002066 | 0.048283 |
| ANKS1B | 66.63653 | -1.12081 | 0.363759 | -3.08118 | 0.002062 | 0.048283 |
| VSIG4 | 749.7111 | 1.014739 | 0.329829 | 3.076563 | 0.002094 | 0.048431 |
| OR2H2 | 10.26756 | -1.21852 | 0.395976 | -3.07726 | 0.002089 | 0.048431 |
| CCDC169 | 62.50541 | -1.13163 | 0.36779 | -3.07684 | 0.002092 | 0.048431 |
| CLDN3 | 467.5857 | -1.42323 | 0.462999 | -3.07393 | 0.002113 | 0.048611 |
| C12orf40 | 5.99017 | -1.64405 | 0.534774 | -3.07429 | 0.00211 | 0.048611 |
| FAM189A1 | 36.33236 | 1.253628 | 0.409168 | 3.063846 | 0.002185 | 0.04971 |
