# Supplementary material for: A novel mitochondrial metabolism-related gene signature for predicting the prognosis of oesophageal squamous cell carcinoma
Source: Aging (Albany NY). 2024 Jun 5;16(11):9649–79. doi: 10.18632/aging.205892 (PMC11210263; doi:10.18632/aging.205892)
Supplement: Supplementary Tables 7-9 [file aging-16-205892-s006.pdf]

Supplementary Table 7. GO and KEGG enrichment analysis results of DEGs.

ONTOLOGY	ID	Description	GeneRatio	BgRatio	p-value	p.adjust	q-value
BP	GO:0035082	axoneme assembly	9/337	87/18800	2.58E-05	0.041122402	0.038946937
BP	GO:0051321	meiotic cell cycle	16/337	266/18800	2.64E-05	0.041122402	0.038946937
CC	GO:0097060	synaptic membrane	19/365	373/19594	8.01E-05	0.019777997	0.017487913
CC	GO:0099699	integral component of synaptic membrane	11/365	149/19594	0.000112495	0.019777997	0.017487913
CC	GO:0005581	collagen trimer	8/365	86/19594	0.000201205	0.019777997	0.017487913
CC	GO:0099240	intrinsic component of synaptic membrane	11/365	160/19594	0.000210965	0.019777997	0.017487913
CC	GO:0062023	collagen-containing extracellular matrix	19/365	429/19594	0.000472793	0.035459497	0.03135366
CC	GO:0043025	neuronal cell body	20/365	482/19594	0.000765607	0.042886688	0.037920861
CC	GO:0043186	P granule	4/365	25/19594	0.001099257	0.042886688	0.037920861
CC	GO:0045495	pole plasm	4/365	25/19594	0.001099257	0.042886688	0.037920861
CC	GO:0060293	germ plasm	4/365	25/19594	0.001099257	0.042886688	0.037920861
CC	GO:0033391	chromatoid body	3/365	12/19594	0.001244723	0.042886688	0.037920861
CC	GO:0043679	axon terminus	8/365	113/19594	0.00125801	0.042886688	0.037920861
CC	GO:0042734	presynaptic membrane	9/365	143/19594	0.001462779	0.044367549	0.039230254
CC	GO:0099056	integral component of presynaptic membrane	6/365	67/19594	0.001538075	0.044367549	0.039230254
KEGG	hsa04080	Neuroactive ligand-receptor interaction	18/157	362/8164	0.000192278	0.044800794	0.043717964

Supplementary Table 8. GSEA analysis of TCGA-ESCC.

ID	setSize	enrichmentScore	NES	p-value	p.adjust	q-value
WP_IL18_SIGNALING_PATHWAY	266	0.424215445	2.132058722	1.00E-10	1.92E-08	1.61E-08
KEGG_OXIDATIVE_PHOSPHORYLATION	113	0.415750078	1.845639559	2.60E-05	0.001010257	0.000848607
REACTOME_METABOLISM_OF_POLYAMINES	58	0.509149923	1.970004759	4.99E-05	0.001612593	0.001354564
WP_ELECTRON_TRANSPORT_CHAIN_OXPHOS_SYSTEM_IN_MITOCHONDRIA	89	0.42884856	1.811634293	0.000100966	0.002762688	0.002320634
REACTOME_THE_CITRIC_ACID_TCA_CYCLE_AND_RESPIRATORY_ELECTRON_TRANSPORT	165	0.310160102	1.417800478	0.00436255	0.049829122	0.041856042
REACTOME_EUKARYOTIC_TRANSLATION_ELONGATION	93	0.655696748	2.794520902	1.00E-10	1.92E-08	1.61E-08
KEGG_RIBOSOME	87	0.652148161	2.753509857	1.00E-10	1.92E-08	1.61E-08
REACTOME_SRP_DEPENDENT_COTRANSLATIONAL_PROTEIN_TARGETING_TO_MEMBRANE	113	0.615279554	2.731410875	1.00E-10	1.92E-08	1.61E-08
REACTOME_EUKARYOTIC_TRANSLATION_INITIATION	120	0.617030923	2.716149663	1.00E-10	1.92E-08	1.61E-08
REACTOME_RESPONSE_OF_EIF2AK4_GCN2_TO_AMINO_ACID_DEFICIENCY	102	0.589136754	2.558808696	1.00E-10	1.92E-08	1.61E-08
REACTOME_SIGNALING_BY_THE_B_CELL_RECEPTOR_BCR	109	0.565117207	2.533317502	1.00E-10	1.92E-08	1.61E-08
REACTOME_NONSENSE_MEDIATED_DECAY_NMD	116	0.549081018	2.438119496	1.00E-10	1.92E-08	1.61E-08
REACTOME_REGULATION_OF_EXPRESSION_OF_SLITS_AND_ROBOS	171	0.503232522	2.306612557	1.00E-10	1.92E-08	1.61E-08
REACTOME_OLFACTORY_SIGNALING_PATHWAY	98	-0.695478407	-2.251386071	1.00E-10	1.92E-08	1.61E-08
KEGG_OLFACTORY_TRANSDUCTION	115	-0.679815158	-2.228880628	1.00E-10	1.92E-08	1.61E-08

Supplementary Table 9. List of miRNAs related key genes.

mRNA	miRNA
COX10	hsa-miR-4533
COX10	hsa-let-7c-3p
COX10	hsa-miR-6809-3p
COX10	hsa-miR-765
COX10	hsa-miR-6124
COX10	hsa-miR-7110-5p
COX10	hsa-miR-5681b
COX10	hsa-miR-11181-3p
COX10	hsa-miR-1343-5p
COX10	hsa-miR-939-5p
COX10	hsa-miR-4492
ACADVL	hsa-miR-5582-5p
ACADVL	hsa-miR-302e
ACADVL	hsa-miR-124-3p
ACADVL	hsa-miR-506-3p
IDH3B	hsa-miR-4447
IDH3B	hsa-miR-4472
IDH3B	hsa-miR-4533
IDH3B	hsa-miR-324-3p
LIAS	hsa-miR-335-3p
LIAS	hsa-miR-3913-5p
LIAS	hsa-miR-3122
LIAS	hsa-miR-1302
LIAS	hsa-miR-4635
LIAS	hsa-miR-2114-5p
LIAS	hsa-miR-892c-5p
LIAS	hsa-miR-493-3p
LIAS	hsa-miR-4503
LIAS	hsa-miR-4298
LIAS	hsa-miR-5010-3p
LIAS	hsa-let-7g-3p
LIAS	hsa-let-7a-2-3p
LIAS	hsa-miR-4679
LIAS	hsa-miR-548b-3p
NDUFB8	hsa-miR-4682
NDUFB8	hsa-miR-513c-3p
NDUFB8	hsa-miR-513a-3p
NDUFB8	hsa-miR-3606-3p
